# Signalling pathway crosstalk stimulated by L-proline drives mouse embryonic stem cells to primitive-ectoderm-like cells

**DOI:** 10.1242/dev.201704

**Published:** 2023-10-26

**Authors:** Hannah J. Glover, Holly Holliday, Rachel A. Shparberg, David Winkler, Margot Day, Michael B. Morris

**Affiliations:** ^1^ School of Medical Sciences, University of Sydney, Sydney 2006, Australia; ^2^ Naomi Berrie Diabetes Center, Columbia Stem Cell Initiative, Department of Pediatrics, Columbia University Irving Medical Center, New York, NY 10032, USA; ^3^ Department of Biochemistry and Chemistry, Latrobe Institute for Molecular Science, Latrobe University, Bundoora 3083, Australia; ^4^ Monash Institute of Pharmaceutical Sciences, Monash University, Parkville 3052, Australia; ^5^ Advanced Materials and Healthcare Technologies, School of Pharmacy, University of Nottingham, Nottingham NG7 2RD, UK

**Keywords:** L-proline, Amino acid, Primitive ectoderm, Cell signalling, Mouse embryonic stem cell, Growth factor

## Abstract

The amino acid L-proline exhibits growth factor-like properties during development – from improving blastocyst development to driving neurogenesis *in vitro*. Addition of 400 μM L-proline to self-renewal medium drives naïve mouse embryonic stem cells (ESCs) to early primitive ectoderm-like (EPL) cells – a transcriptionally distinct primed or partially primed pluripotent state. EPL cells retain expression of pluripotency genes, upregulate primitive ectoderm markers, undergo a morphological change and have increased cell number. These changes are facilitated by a complex signalling network hinging on the Mapk, Fgfr, Pi3k and mTor pathways. Here, we use a factorial experimental design coupled with statistical modelling to understand which signalling pathways are involved in the transition between ESCs and EPL cells, and how they underpin changes in morphology, cell number, apoptosis, proliferation and gene expression. This approach reveals pathways which work antagonistically or synergistically. Most properties were affected by more than one inhibitor, and each inhibitor blocked specific aspects of the naïve-to-primed transition. These mechanisms underpin progression of stem cells across the *in vitro* pluripotency continuum and serve as a model for pre-, peri- and post-implantation embryogenesis.

## Teaser

**Summary:** L-proline acts as growth factor to modulate phosphorylation of the Mapk, Pi3k, Fgf and mTor signalling pathways to drive embryonic stem cells to primitive ectoderm-like cells.

## INTRODUCTION

Amino acids are present in the high micromolar to millimolar range in mammalian reproductive fluid (Aguilar and Reyley, 2005; Cetin et al., 2005; Harris et al., 2005) and are necessary to support normal embryo development *in vivo* (Van Winkle, 2001; Van Winkle et al., 2006; Bazer et al., 2015). Consequently, supplementation of culture media with selected amino acids or certain groups of amino acids can be used to improve preimplantation development (Gardner and Lane, 1993; Lane and Gardner, 1997; Harris et al., 2005). For example, L-proline is a conditionally non-essential amino acid present in tubal fluid at ∼140 µM in mice, ∼150 µM in humans, ∼100 µM in rabbits, 50-300 µM in sheep and ∼200 µM in cows (Aguilar and Reyley, 2005; Cetin et al., 2005). *In vitro*, L-proline improves bovine oocyte maturation rates (Bahrami et al., 2019), promotes development to the blastocyst stage in the mouse system when added during fertilisation (Treleaven et al., 2021) and improves development when added to mouse embryo culture following fertilisation (Morris et al., 2020).

Pluripotent mouse embryonic stem cells (ESCs) serve as an *in vitro* model of mammalian embryo development. When L-proline is added, either in purified form or as part of HepG2 conditioned medium (MEDII), it stimulates cells to transition to a second distinct pluripotent population known as early primitive ectoderm-like cells (EPL cells; Rathjen et al., 1999; Washington et al., 2010) or proline-induced cells (PiCs; Casalino et al., 2011; Comes et al., 2013; D'Aniello et al., 2015, 2017; Patriarca et al., 2021; Cermola et al., 2021; Minchiotti et al., 2022). EPL cells/PiCs are a metastable primed pluripotent population which can revert to naïve ESCs upon removal of L-proline (Rathjen et al., 1999; Washington et al., 2010; Casalino et al., 2011).

The transition to EPL cells recapitulates many of the features of the conversion of inner cell mass (ICM) cells in the 4.5 days post coitum (dpc) mouse embryo to pluripotent primitive ectoderm at ∼5.5 dpc. The primitive ectoderm is now primed to gastrulate and form the three multipotent germ layers (Snow, 1977; Washington et al., 2010; Rivera-Pérez and Hadjantonakis, 2015). The similarities include the following: EPL cells are more prone to exit pluripotency than ESCs and represent a primed or partially primed pluripotent population (Smith, 2017; Hoogland and Marks, 2021; Wang et al., 2021; Glover et al., 2022); expression of the ICM marker *Rex1* (also known as *Zfp42*) in EPL cells is reduced; and there is increased expression of the primitive ectoderm markers *Fgf5* and *Dnmt3b* (Rathjen et al., 1999; Washington et al., 2010; Glover et al., 2022). Colonies undergo a change in morphology, from round and domed to flattened monolayers, and cell-cycle time is reduced from 11 h to 8 h (Stead et al., 2002; Washington et al., 2010; Glover et al., 2022). The continued presence of L-proline in culture primes EPL cells for differentiation to neural cells through a series of embryologically-relevant intermediate cell types (Rathjen et al., 2002; Shparberg et al., 2019a,b; Cermola et al., 2021).

In ESCs, L-proline is taken up via the Snat2 (Slc38a2) transporter (Tan et al., 2011). The mechanisms by which L-proline stimulates development and the transition along the pluripotency continuum include: acute activation of signalling pathways, epigenetic remodelling and regulation of intracellular metabolism (Washington et al., 2010; Casalino et al., 2011; Comes et al., 2013; D'Aniello et al., 2015; 2017; Tan et al., 2016). Collectively, these mechanisms modify a range of emergent properties that drive developmental progression (Washington et al., 2010) and are consistent with L-proline behaving as a growth factor (Morris et al., 2020).

In the mouse embryo, the mTorc1 pathway is required for L-proline-mediated improvement in preimplantation development. L-proline also activates the Erk1/2 and Akt pathways during this time (Morris et al., 2020). When added to ESCs, L-proline acutely activates the same signalling pathways (Lonic, 2006; Washington et al., 2010), as well as the p38 pathway (Tan et al., 2016). Selective inhibition of mTorc1 (with rapamycin) or Mek1/Erk1/2 (with U0126) or P38 (with SB203580 or PP2) prevents upregulation of the EPL cell marker *Dnmt3b* (Lonic, 2006; Washington et al., 2010). Conversely, inhibition of the Pi3k/Akt pathway with LY294002 blocks the morphology change and increase in proliferation but allows the associated gene-expression changes to occur (Lonic, 2006). Thus, several signalling pathways are involved in the transition from ESCs to EPL cells. Selective inhibition of these pathways blocks different aspects of the transition, and collectively this shows that L-proline modulates a complex signalling network. These experiments did not comprehensively measure changes in a range of emergent properties or expression of marker genes required to better understand this complex network.

To explore this, we employed inhibitors of Mek1 (Map2k1), Fgf receptor (Fgfr), Pi3k, mTorc1 and P70-S6 kinase (S6k) individually and in combinations. The results of these factorial experiments were analysed with supervised learning and unsupervised machine learning models including kmeans clustering, multiple linear regression (MLR), MLR with interaction terms and Bayesian regularised neural network with a Gaussian prior (BRANNGP; Woolf et al., 2005; Burden and Winkler, 2008; Winkler and Burden, 2012; Epa et al., 2013). MLR with interaction terms was used to calculate synergistic and antagonistic effects. This approach is most commonly used to determine drug interactions (Sorokin et al., 2018; Julkunen et al., 2020; Panina et al., 2020) and is becoming increasingly used in stem cell biology (Chang and Zandstra, 2004; Prudhomme et al., 2004; Audet, 2010; Jakobsen et al., 2014; Ireland et al., 2020).

## RESULTS

### ESC-to-EPL cell transition alters gene expression and emergent properties

**Fig. 1.**
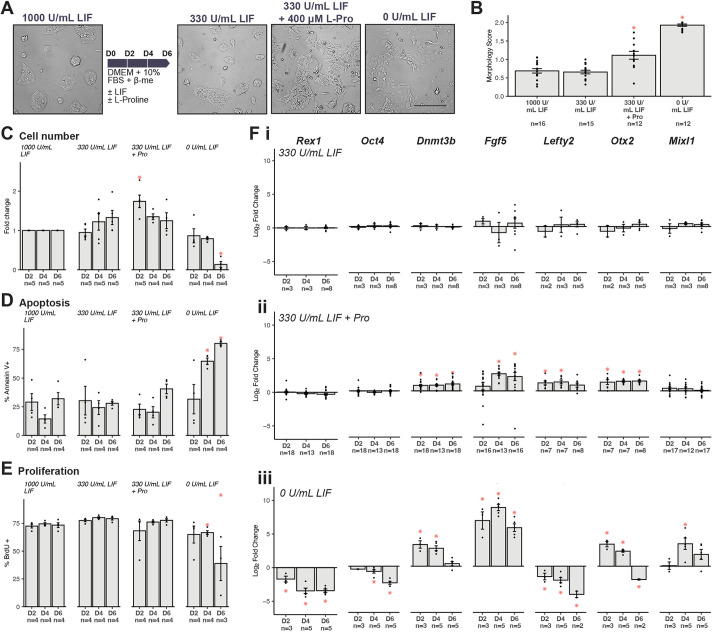
**L-proline drives ESCs to EPL cells.** (A) Representative images showing ESCs that have self-renewed in medium containing 1000 U/ml LIF or were cultured in medium containing 330 U/ml LIF, 330 U/ml LIF+L-proline or no LIF for 6* *days. Scale bar: 100 μm. Note that the representative micrographs are the same as those shown in Fig. 5. (B) Colony morphology was scored at day 6. (C-E) Cell number (C), apoptosis (D) and proliferation (E) were measured at days 2, 4 and 6. (F) At days 2, 4 and 6, changes in expression of pluripotency genes (*Rex1* and *Oct4*), primitive ectoderm markers (*Dnmt3b*, *Fgf5*, *Lefty2* and *Otx2*) and mesendoderm genes (*Mixl1*) in cells grown in medium containing 330 U/ml LIF (i), 330 U/ml LIF+L-proline (ii) and no LIF or L-proline (iii) were measured. All samples were normalised to *Actb* and then to cells grown in 1000 U/ml LIF. All graphs (B-F) show mean±s.e.m. with individual data points. Data were analysed using one-way ANOVA with Dunnett's multiple comparisons test by condition, comparing with cells grown in 1000 U/ml LIF. *P<0.05.

ESCs were maintained in either 330 or 1000 U/ml LIF then directed to transition into EPL cells by addition of 400 μM L-proline for 6* *days. In addition, cells were also allowed to undergo spontaneous differentiation without added LIF (Fig. 1A). All ESCs grown in 330 or 1000 U/ml LIF maintained their dome-shaped colonies and exhibited no differences in morphology score. Colony morphology changed significantly to flattened epithelial-like colonies in the presence of L-proline. Cells allowed to differentiate spontaneously underwent a more robust morphology change (Fig. 1A,B), consistent with these cells undergoing differentiation beyond the EPL cell stage (Tan et al., 2011; Minchiotti et al., 2022).

Cell number, apoptosis and proliferation were quantified at days 2, 4 and 6 of culture. Cell number was normalised to cells growing in 1000 U/ml LIF. Cells growing in 330 U/ml LIF+400 μM L-proline increased cell number by 1.7-fold at day 2 (Fig. 1C), consistent with previous results for ESCs grown in 1000 U/ml LIF+L-proline (Washington et al., 2010). No significant changes in proliferation or apoptosis were observed (Fig. 1D,E). Cells undergoing spontaneous differentiation without LIF or L-proline were dying by day 6, with cell number reduced by 85% (Fig. 1C). Apoptosis increased by 4.2-fold at day 4 and 2.3-fold at day 6 (Fig. 1D). Proliferation decreased by 50% at day 6 (Fig. 1E), suggesting deficiencies in medium formulation and therefore a reduced capacity to support growth of differentiating cells.

After 6* *days of culture, gene expression was profiled, focusing on pluripotency genes *Rex1* and *Oct4* (*Pou5f1*), primitive ectoderm markers *Dnmt3b*, *Fgf5*, *Lefty2* and *Otx2*, and the mesendoderm marker *Mixl1*. There were no differences in the expression of any of the genes between ESCs grown in 330 or 1000 U/ml LIF (Fig. 1Fi). Cells grown in 330 U/ml LIF+400 μM L-proline had comparable expression of *Rex1* and *Oct4*, indicating that maintenance of pluripotency and the expression of the mesendoderm marker *Mixl1* also did not change. The expression of all primitive ectoderm markers increased (Fig. 1Fii). Cells allowed to spontaneously differentiate had a gene expression profile consistent with rapid, unregulated differentiation – reduced expression of *Rex1* and *Oct4* and a transient wave of expression of *Dnmt3b*, *Fgf5* and *Otx2*. The expression of *Lefty2* remained strongly reduced throughout, whereas *Mixl1* expression increased significantly (Fig. 1Fiii).

### L-proline-mediated phosphorylation of signalling pathway intermediates

**Fig. 2.**
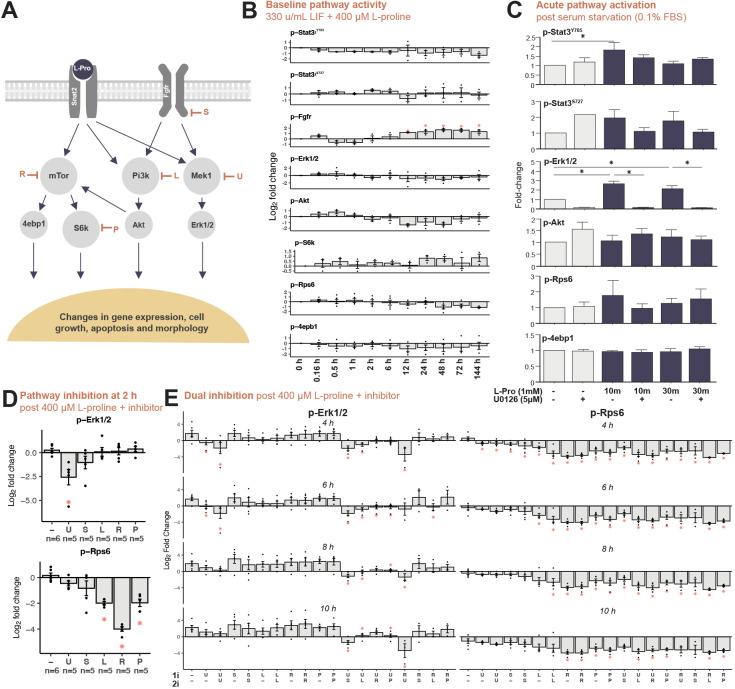
**L-proline acts through Fgfr, Mapk, Pi3k and mTor signalling pathways.** (A) L-proline enters the cell via the Snat2 transporter and activates the Mapk pathway (which can be inhibited by U0126, U), the Pi3k pathway (which can be inhibited by LY294002, L), the mTor pathway (which can be inhibited by rapamycin, R), the downstream mTor kinase S6k (which can be inhibited by PF-4708671, P), or it indirectly activates the Fgfr (which can be inhibited by SU5402, S). Activation or inhibition of these pathways affects both gene expression and emergent cellular properties. (B) Naïve ESCs were grown in medium containing 330 U/ml LIF+400 μM L-proline for up to 6* *days (144 h). Cell lysates were analysed by western blotting for: p-Stat3^Y705^ or p-Stat3^S727^ (*n*=3 each), p-Fgfr^Y653/Y654^ (*n*=3), p-Erk1/2^T202/Y204^ (*n*=4), p-Akt^S473^ (*n*=3), p-S6k^T389^ (*n*=3), p-Rps6^S235/S236^ (*n*=5) and p-4ebp1^T37/T46^ (*n*=4). Data shows quantification of western blot bands. Integrated intensity was calculated for each band and was normalised to β-tubulin and to the 0 h untreated sample. Graphs show log_2_ fold change±s.e.m. and individual data points. Data were analysed using one-way ANOVA with post hoc Dunnett's multiple comparison test to the 0 h sample. (C) Naïve ESCs were serum starved in 0.1% foetal bovine serum for 4 h, with 5 μM U0126 added for the final 30 min. Cells were then left untreated or treated with 1 mM L-proline for 10 or 30 min. All conditions *n*=3. Integrated intensity was calculated for each band and was normalised to t-Erk1/2 and to an untreated serum-starved ESC sample. Graphs show fold change±s.e.m. Data were analysed using one-way ANOVA with post hoc Tukey's multiple comparison test. (D,E) Signalling pathway inhibitors (1i) and 400 μM L-proline were added to naïve ESCs growing in 1000 U/ml LIF at 0 h. After 2 h, cell samples were analysed for p-Erk1/2 and p-Rps6 (D). At 2, 4, 6 or 8 h, a second dose of the same inhibitor or a different inhibitor (2i) was added, and samples were collected 2 h later (E). Cell samples were analysed for p-Erk1/2 and p-Rps6. Data were collected from *n*=3-6 replicates. Integrated intensity was calculated for each band and was normalised to β-tubulin and normalised to an untreated ESC sample. Graphs show log_2_ fold change±s.e.m. and individual data points. Data were analysed using one-way ANOVA with post hoc Dunnett's multiple comparison test to the respective timepoint treated with L-proline only. *P<0.05.

We examined the phosphorylation status of signalling pathway intermediates drawn from the Stat3, Fgf, Mek1/Erk1/2, Pi3k/Akt, mTor, p38 and Pkc pathways (Fig. 2A), each of which is known to play a role in pluripotency and cell state transition of ESCs (Kunath et al., 2007; Lanner and Rossant, 2010; Washington et al., 2010; Cherepkova et al., 2016; Tan et al., 2016).

Naïve, self-renewing ESCs were switched from 1000 U/ml LIF to EPL cell medium containing 330 U/ml LIF+400 μM L-proline and the phosphorylation status of the pathway intermediates was quantified by western blot over the short (0-12 h) and long term (1-6* *days). Only phosphorylation of Fgfr increased modestly but significantly from 12 h onwards (Fig. 2B; Fig. S1A). The P38 and Pkc pathways, including the downstream Hsp27, previously shown to be altered by addition of L-proline (Tan et al., 2016), were not detected in ESCs±L-proline under our conditions (Fig. S1B). After 4 h of serum starvation [Dulbecco's Modified Eagle Medium (DMEM)/0.1% foetal bovine serum (FBS)/β-mercaptoethanol (β-Me) only], treatment with 1 mM L-proline acutely increased phosphorylation of Erk1/2 and Stat3^Y705^ (Fig. 2C; Fig. S1C).

### Signalling pathway inhibition illustrates pathway crosstalk

The effect of signalling pathway inhibitors, individually or in combination, on L-proline-mediated pathway activity was assessed using the following: (1) Mapk pathway using the Mek1/2 inhibitor U0126 (U) (Favata et al., 1998); (2) Fgfr pathway using receptor antagonist SU5402 (S) (Mohammadi et al., 1997); (3) Pi3k/Akt pathway using the Pi3k inhibitor LY294002 (L) (Vlahos et al., 1994); (4) mTorc1 pathway using the mTorc1 complex inhibitor rapamycin (R) (Sabers et al., 1995); (5) mTorc1 pathway at the downstream kinase, S6k, using the inhibitor PF-4708671 (P) (Pearce et al., 2010).

ESCs were cultured under standard conditions (1000 U/ml LIF). At 0 h, 400 μM L-proline was added, along with the first inhibitor (1i). Second doses (2i) of an inhibitor were added 2 h before sample collection. An initial dose of U0126 suppressed L-proline-mediated Erk1/2 (downstream of Mek1) phosphorylation only for 6 h, after which phosphorylation returned to the level seen with L-proline only (Fig. 2D,E; Fig. S1D,E). Furthermore, any second dose of U0126 failed to suppress Erk1/2 phosphorylation for longer than 6 h (Fig. 2E; Fig. S1D,E), suggesting that Erk1/2 phosphorylation was no longer controlled by Mek1.

None of the other four pathway inhibitors suppressed Erk1/2 phosphorylation on its own (Fig. 2E; Fig. S1D,E). However, extended suppression of Erk1/2 phosphorylation occurred up to 10 h when SU5401 or LY294002 or PF-4708671 were added after the initial addition of U0126 (Fig. 2E; Fig. S1D,E), suggesting that Erk1/2 phosphorylation was now controlled by crosstalk involving the Fgfr/Pi3k/S6k axis.

Rps6 phosphorylation showed similar interplay between pathways. Rps6 phosphorylation was suppressed for up to 10 h by rapamycin and up to 8 h by LY294002 (Fig. 2E; Fig. S1D,E), consistent with it modulating the mTorc1/S6k signalling axis (Fig. 2A). However, Rps6 phosphorylation was also temporarily suppressed by inhibition of Mek1, Fgfr and Pi3k, or combinations of Mek1 inhibition followed by Fgfr inhibition, or Mek1 inhibition followed by Pi3k inhibition (Fig. 2E; Fig. S1D,E). These results suggest complex, dynamic changes in signalling pathway activity over time in the presence of L-proline.

### Factorial experiments reveal relationships between emergent properties

**Fig. 3.**
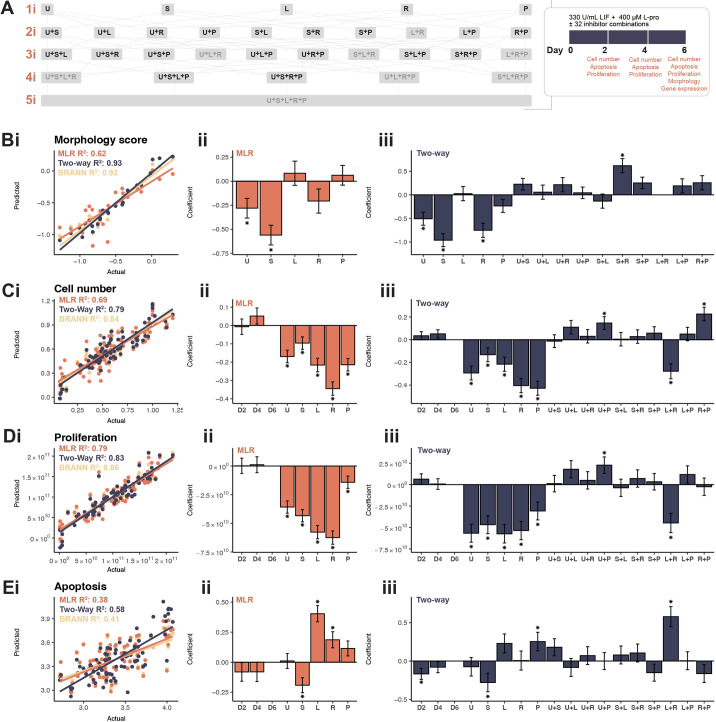
**Signalling pathway inhibitors regulate emergent properties during the ESC-to-EPL cell transition.** (A) A five-level factorial experimental design showing all combinations of the five signalling pathway inhibitors. Naïve ESCs were cultured over 6* *days in 330 U/ml LIF+400 μM L-proline with combinations of five inhibitors (U, U0126; S, SU5402; L, LY294002; R, rapamycin; P, PF-4708671). The eight combinations containing both L and R, for which cells were non-viable from day 2, are shown with greyed-out letters. (B-E) Colony morphology was scored on day 6 (B), whereas cell number (C), proliferation (D) and apoptosis (E) were recorded at days 2, 4 and 6. Data were averaged across biological replicates where *n*≥3. To correct for non-normal distributions, morphology and apoptosis data were log transformed, and proliferation data were raised (x^6^). Data were modelled using either MLR, MLR with two-way interaction terms or a BRANNGP, with scatter of each model comparing the actual fit with the prediction from the model with adjusted R^2^ (i). Coefficients for each variable±s.e.m. for standard MLR (ii). Coefficients for each variable±s.e.m. for MLR with two-way interaction terms (iii). *P<0.05.

To further elucidate pathway interactions and their effect of emergent properties, a factorial experiment was designed to monitor the ESC-to-EPL transition over 6* *days in the presence of all possible combinations of the five inhibitors (Fig. 3A). On days 2 and 4, cells were counted during replating, and samples were collected to quantify apoptosis and cell proliferation by flow cytometry. At day 6, in addition to measurements of apoptosis and cell proliferation, cells were imaged for colony morphology and qPCR was used to quantify changes in the expression of marker genes.

At day 2, the eight conditions containing both LY294002 and rapamycin had poor viability. Cell number had reduced by 90% (Fig. S3), proliferation reduced by nearly 40% (Fig. S4) and apoptosis had increased by more than 50% (Fig. S5). By day 4, very few live cells were present. These conditions were considered to be non-viable and were not considered in the day 4 and 6 measurements.

To get a broader understanding of relationships between emergent properties and gene expression, a correlation matrix was generated using all data from all viable inhibitor combinations (Fig. S6A). Expected correlations were observed, such as: (1) a positive correlation between cell number and proliferation (*r*≤0.57); (2) a negative correlation between cell number and apoptosis (*r*≥−0.43) across the 6* *days of transition to EPL cells; and (3) the coupling of expression of pluripotency markers *Oct4* and *Rex1* (*r*=0.80) and EPL-cell markers *Dnmt3b* and *Fgf5* (*r=*0.52). However, more nuanced correlations were observed, including positive correlations between: (1) proliferation and morphology (*r*=0.31 at day 4 and 0.41 at day 6); and (2) proliferation (at days 4 and 6) and expression of primed pluripotency genes: *Dnmt3b* (*r*≤0.49), *Fgf5* (*r*≤0.32) and *Mixl1* (*r*≤0.50). A high correlation was also observed between expression of the primitive ectoderm marker *Lefty2* and the pluripotency markers *Oct4* (*r*=0.55) and *Rex1* (*r*=0.49).

These relationships were further explored using principal component analysis (PCA) with kmeans clustering. Two clusters were identified based on the silhouette score (Fig. S7A). The first cluster included the EPL cell control (Fig. S7B) and contained the highest proportion of conditions with LY294002 (Fig. S7C). This cluster most closely resembled EPL cells, including a higher morphology score, cell number and proliferative rate, and higher expression of primed pluripotency genes *Dnmt3b*, *Fgf5* and *Lefty2* (Fig. S7D). The second cluster appeared to be more ESC-like and included a high proportion of conditions with SU5402 and rapamycin (Fig. S7C).

### Data modelling helps deconvolute complex signalling networks

To understand which signalling pathways or pathway combinations drive changes in gene expression and emergent properties, we employed supervised learning techniques to generate MLR and BRANNGP models (Figs 3Bi-Ei, 4Ai-Gi).

**Fig. 4.**
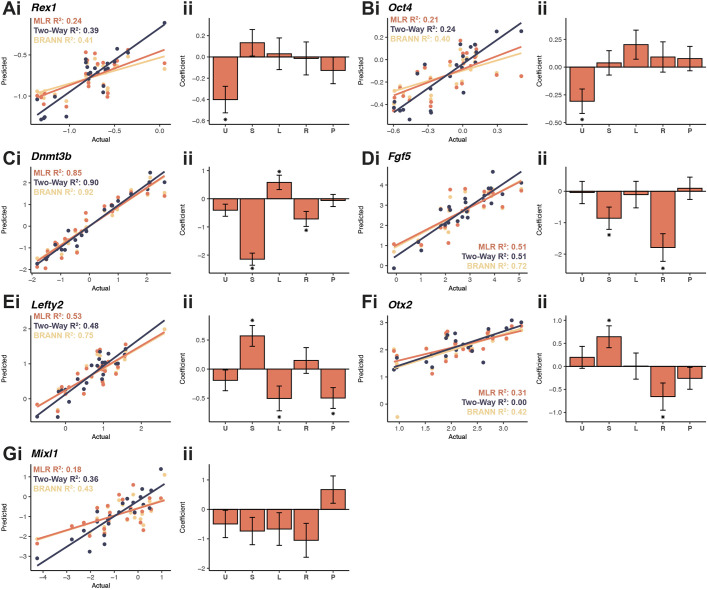
**Signalling pathway inhibitors regulate gene expression during the ESC-to-EPL cell transition.** (A-G) Naïve ESCs were cultured over 6* *days in 330 U/ml LIF+400 μM L-proline with combinations of five inhibitors (U, U0126; S, SU5402; L, LY294002; R, rapamycin; P, PF-4708671). Quantification of changes in expression of pluripotency genes *Rex1* (A) and *Oct4* (B), primitive ectoderm markers *Dnmt3b* (C), *Fgf5* (D), *Lefty2* (E) and *Otx2* (F), and mesendoderm gene *Mixl1* (G) at day 6. All samples were normalised to *Actb* and then to cells grown in 1000 U/ml LIF. Data were averaged across biological replicates where *n*≥3. Data were modelled using either MLR, MLR with two-way interaction terms or a BRANNGP, with scatter of each model comparing the actual fit with the prediction from the model with adjusted R^2^ (i). Coefficients for each variable±s.e.m. for standard MLR (ii). *P<0.05.

#### MLR models

MLR models are a supervised learning approach used to understand linear relationships and to assign the relative importance of factors and their interactions. MLR models are easy to interpret but are less accurate at predicting the responses to multiple inhibitors when the underlying relationships are nonlinear. The coefficients of the MLR models denote the sign and magnitude of the contribution each inhibitor makes to the responses (Figs 3Bii-Eii, 4Aii-Gii). In addition to standard MLR, we also generated MLR models with two interaction terms to determine whether inhibitors were acting independently (additive), synergistically or antagonistically (Fig. 3Biii-Eiii). For these models, the coefficients for the inhibitors alone were compared with those for the interaction term. An additive effect occurs when the response to multiple inhibitors is the sum of the individual contributions of the inhibitors; i.e., no significant interaction effect. A synergistic effect occurs where the interaction terms enhance the response more than the sum of the individual contributions of the inhibitors. An antagonistic effect occurs where the interaction terms reduce the response below the sum of the individual contributions of the inhibitors. The most robust and predictive MLR models have higher adjusted R^2^ and lower standard error (σ) values (Table S1). An *F*-test was used to determine whether MLR models with increasing numbers of parameters (e.g. a two-way versus a one-way MLR) resulted in a significantly improved fit (Table S2).

#### Nonlinear neural network models

These machine learning models are used to capture nonlinearity and interactions in stimulus-response relationships. They predict the responses of cells to new combinations of factors more accurately than MLR models but are harder to interpret, so their most important use is in more accurately predicting the effect of different factors in stem cell behaviour. They are therefore complementary to linear MLR models. We used BRANNGP to assess nonlinearity and to create models without issues of overfitting and overtraining (Burden and Winkler, 2008; Winkler and Burden, 2012). This algorithm automatically optimises model complexity. Most datasets had substantially improved adjusted R^2^ values when modelled using BRANNGP (Table S1), reinforcing the fact that nonlinearity and/or interaction were important. The average adjusted R^2^ value for BRANNGPs was 0.64±0.07 compared with 0.48±0.07 for MLR models and 0.53±0.09 for MLR models with two-way interaction terms. Only apoptosis and proliferation models had adjusted R^2^ values for BRANNGP models similar to those for the MLR models, suggesting that the relationship was approximately linear. The improved predictions of the neural network model for the other datasets imply that nonlinearity or interactions between factors are important for these biological endpoints. In nonlinear models, the importance of model features are local, rather than global, so they depend on where they are assessed on the response surface defined by the nonlinear model (Rengasamy, et al., 2022). Thus, the nature and magnitude of these factors cannot be as readily deconvoluted from the BRANNGP models.

### Morphology is regulated by Erk1/2, Fgfr and mTor

The morphology dataset (Fig. S2) was used to train MLR, MLR with two-way interaction terms and BRANNGP models (Fig. 3Bi). The MLR model with no interaction terms showed that addition of SU5402 or U0126 prevented changes in colony morphology normally expected in the presence of L-proline (Fig. 3Bii), resulting in cells which retained a domed, ESC-like appearance. An *F*-test indicated that MLR with two-way interaction terms provided a better fit than the MLR (Table S2). This improved model showed that SU5402, U0126 and rapamycin all prevent morphology change (Fig. 3Biii). There was no significant interaction between SU5402 and U0126, indicating that this effect was largely additive. There was a significant interaction between SU5402 and rapamycin mediating morphology change. This interaction coefficient was reversed, indicating an antagonistic effect.

### All inhibitors decrease cell number and proliferation

Modelling was generated for cell number and proliferation data from all inhibitor combinations. MLR produced robust fits for both cell number and proliferation with adjusted R^2^ of 0.69 and 0.79 respectively (Fig. 3Ci,Di). All inhibitors significantly reduced cell number and proliferation, with rapamycin having the largest effect (Fig. 3Cii,Dii).

For both cell number and proliferation, the MLR with two-way interactions had an improved adjusted R^2^ (0.79 and 0.83, respectively), and the *F*-test showed significant improvement (Table S2). The individual effects were retained for each inhibitor, but multiple interaction effects were noted: (1) U0126 and PF-4708671 were antagonistic in both cell number and proliferation models; (2) antagonism between rapamycin and PF-4708671 in the cell number model; (3) LY294002 and rapamycin were strongly synergistic for both cell number and proliferation models (Fig. 3C-Diii). An alternate model, which attempts to overcome non-normality of the proliferation input data by dividing the data into octiles, exhibited very similar results to the MLR model (Fig. S8).

### Apoptosis is differentially altered by each inhibitor

Apoptosis data generated an adequate fit using the MLR (adjusted R^2^ of 0.38), but this was significantly increased using the MLR with two-way interactions (adjusted R^2^ of 0.58 and *F*-test *P*<0.05; Fig. 3Ei; Table S2). In the MLR model without interaction terms, SU5402 reduced apoptosis and LY294002 and rapamycin each increased apoptosis (Fig. 3Eii). In the MLR with two-way interactions, the individual effects for LY294002 and rapamycin were lost, and instead there was a strong synergistic effect. The day 2 parameter was also significant in the model (Fig. 3Eiii). In conjunction with the reduction in proliferation, this explains the early cellular lethality of the combination of LY294002 and rapamycin, which does not occur when the inhibitors are used individually.

The apoptosis model highlighted differential effects on apoptosis between SU5402 and PF-4708671 (Fig. 3Eiii). The increase in apoptosis in PF-4708671-treated cells combined with the decrease in proliferation (Fig. 3Diii) underpins the net decrease in cell number (Fig. 3Ciii). Contrarily, SU5402-treated cells had reduced apoptosis combined with decreased proliferation (Fig. 3Diii) resulting in a net decrease cell number (Fig. 3Ciii), indicating that proliferation was the main driver of decreased cell number. U0126 did not affect apoptosis in the presence of L-proline (Fig. 3Eii), indicating that the decrease in cell number elicited by U0126 is due entirely to a decrease in proliferation (Fig. 3Cii,Dii).

### Gene expression is regulated by intracellular signalling

Computational modelling was also used to assess how inhibitors impacted gene expression at day 6 (Fig. 4; Fig. S9). The MLR model for *Dnmt3b*, *Fgf5* and *Lefty2* had a robust fit (adjusted R^2^>0.5, Fig. 4Ci-Ei). Models for *Rex1*, *Oct4* and *Otx2* expression had a modest fit (adjusted R^2^ of 0.24, 0.21 and 0.31, respectively; Fig. 4Ai,Bi,Fi). None of these models was improved significantly by adding two-way interaction effects (*F*>0.05; Table S2; Fig. S10).

The MLR model showed that U0126 decreased expression of the pluripotency genes *Rex1* and *Oct4* (Fig. 4Aii,Bii). The decrease was modest when compared with the strong downregulation of these genes for cells undergoing spontaneous differentiation (Fig. 1Fiii).

Inconsistent changes in expression patterns occurred frequently for the EPL markers *Dnmt3b*, *Fgf5*, *Lefty2* and *Otx2.* For example, SU5402 decreased expression of *Dnmt3b* and *Fgf5* (Fig. 4Cii,Dii) but increased expression of *Lefty2* and *Otx2* (Fig. 4Eii,Fii). LY294002 increased *Dnmt3b* expression (Fig. 4Cii) but reduced that of *Lefty2* (Fig. 4Eii). Rapamycin decreased *Dnmt3b*, *Fgf5* and *Otx2* expression (Fig. 4Cii,Dii,Fii) but did not alter that of *Lefty2*. PF-4708671 only reduced *Lefty2* expression (Fig. 4Eii). These results highlight that expression of individual genes associated with the identity of EPL cells are associated with complex signalling pathway control. In all cases, two or three of the pathways we probed regulated expression of each gene. The MLR models for the mesendoderm gene *Mixl1* were not sufficiently statistically significant to be able to make strong biological statements about this gene (adjusted R^2^ of 0.18; Fig. 4Gi).

### Functional assays establish where inhibitor-treated cells fall on the pluripotency continuum

The complexity of how inhibitors drive expression of EPL marker genes was further exemplified by data showing that many inhibitor combinations suppressed some but not all primitive ectoderm genes (Fig. S9). We ran a functional assay to identify the pluripotency capacity of each inhibitor combination. After 6* *days of culture in L-proline and the various inhibitors, cells were allowed to spontaneously differentiate as embryoid bodies (EBs). Samples were collected on days 2, 3 and 4 and qRT-PCR was used to quantify expression of the primitive streak marker brachyury (*T*). In the absence of inhibitors, more naïve cells – like ESCs – took 4* *days to upregulate *T* expression*,* whereas more primed cells – like EPL cells – upregulated expression of *T* at day 2 (Fig. S11A). Across all inhibitor experiments, conditions that contained U0126 or LY294002 tended to upregulate *T* expression earlier, and conditions that contained rapamycin tended to upregulate *T* later (Fig. S11Bii). We also assessed the correlations between the slope of *T* upregulation and other genes. Significant positive correlations were found between *T* upregulation and *Dnmt3b*, *Fgf5* and *Mixl1*, but not the pluripotency markers *Rex1* and *Oct4* or the more recently adopted primitive ectoderm markers *Lefty2* and *Otx2* (Fig. S6B).

## DISCUSSION

### Signalling pathways active during the ESC-to-EPL transition

**Fig. 5.**
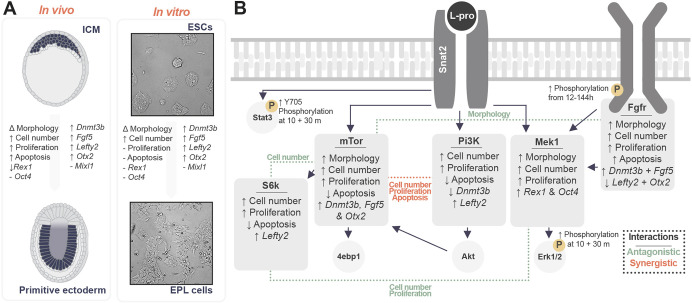
**Summary of signalling pathway-mediated changes in emergent properties and gene expression during the ESC-to-EPL cell transition.** (A) The L-proline-mediated ESC-to-EPL cell transition recapitulates the transition from the inner cell mass (ICM) to the primitive ectoderm (Snow, 1977; Coucouvanis and Martin, 1999; Brennan et al., 2001; Hart et al., 2002; Pelton et al., 2002; Watanabe et al., 2002; Acampora et al.,, 2013). Note that the representative micrographs are the same as those shown in Fig. 1. (B) Results of MLR show that L-proline acts through each signalling pathway to control different aspects of cell state transition. Interaction effects were noted between some inhibitor combinations, and these are shown in dotted lines, with red dotted lines denoting synergistic effects, where two inhibitors produce a response larger than either alone, and green dotted lines denoting antagonistic effects, where two inhibitors produce a response less than the sum of either alone.

The small molecule inhibitors used in this study helped elucidate the role of various signalling pathways that mediate self-renewal, cell state transitions, differentiation and other emergent properties such as colony morphology, cell number, proliferation and apoptosis during the transition from ESCs to EPL cells (Fig. 5A), namely the Mapk (using the Mek1 inhibitor U0126), Fgfr (using the antagonist SU5402), Pi3k (using the Pi3k inhibitor LY294002) and mTor (using the mTorc1 complex inhibitor rapamycin, or the S6k inhibitor PF-4708671) pathways. These signalling pathways are acutely activated by L-proline (Fig. 2B,C) or have been previously associated with L-proline-mediated formation of EPL cells (Lonic, 2006; Washington et al., 2010; Tan et al., 2016).

In the absence of inhibitors, L-proline increased pathway phosphorylation (Fig. 5B), including acute phosphorylation of Stat3^Y705^ and Erk1/2 within 10 min (Fig. 2C; Fig. S1C). This suggests that L-proline can rapidly induce changes in pathways known to be important for maintenance or loss of pluripotency (Stavridis et al., 2010; Huang et al., 2014). Over 6 days, Fgfr phosphorylation increased but there was no change in the canonical intermediate Erk1/2 (Fig. 2B; Fig. S1A), suggesting that Fgfr is signalling through other intermediates such as Pkc, Pi3k, Src, Stat1, P38 and Jnk (Dailey et al., 2005).

When signalling pathway inhibitors were used in the presence of L-proline, signalling pathway crosstalk led to maintenance of Mapk signalling: Erk1/2, immediately downstream of U0126 target Mek1, had decreased phosphorylation in the presence of this inhibitor but only for 6 h. A second dose of U0126 did not extend this time (Fig. 2E; Fig. S1D,E). This effect is not unique to U0126. Erk1/2 phosphorylation is only transiently reduced when a variety of Mek1 inhibitors (PD98059, PD184352, PD0325901 and U0126) are added to the culture medium of ESCs (Chen et al., 2015). Together these results argue against the loss of U0126 activity, but rather that Erk1/2 phosphorylation is maintained by pathway crosstalk which bypasses Mek1. As reduced phosphorylation of Erk1/2 in the presence of U0126 could be extended to 10 h by adding the Fgfr inhibitor SU5402 or the inhibitor LY294002, it is plausible that the Fgfr-Pi3k-Akt axis sustains L-proline-mediated phosphorylation of Erk1/2 (Dailey et al., 2005). This complex network with multiple inputs speaks to the importance of cells maintaining Erk1/2 phosphorylation to avoid widespread apoptosis, as seen in *Erk1^−/−^/Erk2^−/−^* ESCs (Chen et al., 2015).

### Modelling reveals inhibitors that modulate the transition of ESCs to EPL cells

Our factorial study assessed how signalling pathways influence a variety of properties during the L-proline-mediated transition from ESCs to EPL cells (Fig. 5). No single inhibitor was sufficient to explain all the changes in gene expression and emergent properties during ESC transition to EPL cells. Rather, our modelling suggests that these signalling pathways have discrete roles within this transition, likely supported by signalling pathway crosstalk.

The combinational experiments revealed the following information for each pathway inhibitor: (1) When the Mapk/Erk1/2 pathway was inhibited by U0126, cells did not undergo the morphological change associated with the presence of L-proline (Fig. 3Aii), despite a decrease in *Rex1* and *Oct4* expression (Figs 4A-B, 5). This decrease was less than in cells undergoing spontaneous differentiation (Fig. 1Fiii), indicating disruption of the pluripotency gene regulatory network (Kim et al., 2008). U0126 did not alter expression of any of the primitive ectoderm markers (Fig. 4C-F). (2) When Fgfr was inhibited by SU5402, cells did not undergo a morphology change (Fig. 3Bii) and expression of the EPL-cell markers *Dnmt3b* and *Fgf5*, which is increased in the presence of L-proline alone, was blocked. In contrast, expression of EPL-cell markers *Otx2* and *Lefty2* increased in the presence of this inhibitor (Figs. 4C-F, 5). This suggests that Fgfr inhibition at least partially blocks the transition. (3) When the Pi3k/Akt pathway was inhibited with LY294002, the L-proline-mediated change in colony morphology still occurred, as did the increased expression of EPL-cell markers *Dnmt3b* and *Fgf5*. The L-proline-mediated increase in *Otx2* expression also occurred, but L-proline-mediated increase in *Lefty2* expression was suppressed. An early increase in *Lefty2* expression is associated with the transition of ESCs to EPL cells, but *Lefty2* expression is low when cells undergo lineage restriction (Harvey et al., 2010). These results suggest that increased *Lefty2* expression is not obligatory for the transition. (4) When mTorc1 was inhibited by rapamycin, ESCs underwent L-proline-mediated change in morphology, but increased expression of *Dnmt3b*, *Fgf5* and *Otx2* was suppressed. This suppression is consistent with previously published data (Washington et al., 2010). However, in that study rapamycin blocked the morphology change, which we did not observe. Earlier protocols generated EPL cells using 1000 U/ml LIF and L-proline, which inconsistently upregulated expression of the primitive ectoderm marker *Fgf5* (Washington et al., 2010). Here, we reduced LIF to 330 U/ml, which provided robust upregulation of *Fgf5* expression (Harvey et al., 2010; Glover et al., 2022). These results highlight the sensitive balance between the cytokine LIF and the growth-factor-like properties of L-proline in promoting directed cell state transitions. (5) When the S6k branch of the mTorc1 pathway was inhibited with PF-4708671, like with rapamycin, it failed to prevent the L-proline-mediated change in colony morphology. However, unlike rapamycin it did not suppress the L-proline-mediated increase in the expression of the EPL-cell markers *Dnmt3b*, *Fgf5* and *Otx2* (Figs 4C-D,F, 5). This suggests that stimulation of expression of these markers by L-proline requires the 4ebp1 (Eif4ebp1) branch of the mTorc1 pathway.

All five inhibitors reduced cell number and reduced the rate of proliferation compared with L-proline (Fig. 3Cii,Ciii,Dii,Diii) but different effects were seen on apoptosis (Fig. 3Eii,Eiii). Cells treated with LY294002 or rapamycin each significantly increased apoptosis in the MLR model, but individual effects were lost in the MLR with interaction terms in favour of the strong synergistic effect. This strong synergistic effect is also seen in the proliferation and cell number models and underpins the lack of cell viability in conditions containing LY294002 and rapamycin. These results are consistent with Pi3k being a strong mediator of cell survival and progression (Chang et al., 2003; Takahashi et al., 2005; Tsurutani et al., 2005; Yu and Cui, 2016).

The synergistic effect of LY294002 and rapamycin on cell death was not seen in interactions with other the other mTOR-associated inhibitor, PF-4708671, which blocks the S6k branch of the mTorc1 pathway. Rather, the combination of LY294002 and PF-4708671 had only an additive effect and the combination of rapamycin and PF-4708671 had and antagonistic effect. Both results support the hypothesis that mTorc1 signalling via the 4ebp1 branch is anti-apoptotic (Nawroth et al., 2011; Pons et al., 2011; Yellen et al., 2011) and pro-proliferative (Dowling et al., 2010; Nawroth et al., 2011).

Inhibition of the Mek1/Erk1/2 pathway with U0126 did not affect apoptosis, and the inhibition of Fgfr with SU5402 decreased apoptosis. These conditions had a net reduction in cell number, driven by reduced proliferation. Fgfr signalling produces cell- and state-specific effects on apoptosis and proliferation (Dailey et al., 2005), and the reduced apoptosis observed with SU5402 provides further support for this.

Collectively, these results highlight biological system complexity and make it difficult, if not impossible, *a priori* to determine outcomes even when a single inhibitor is used. These pathways could be better elucidated by phosphoproteomics using mass spectrometry (Li et al., 2011) to identify additional signalling pathways that may be involved, including the recently identified L-proline target Tgf-β (D'Aniello et al., 2017). These pathways can be functionally addressed using automated high throughput factorial screening for key predictive properties such as morphology or proliferation, which were shown to have high positive correlations with EPL markers *Dnmt3b* and *Fgf5* (Fig. S6A).

### Primitive ectoderm markers reflect spatial and temporal contributions to the EPL-cell transition

We selected four primitive ectoderm markers, *Dnmt3b*, *Fgf5*, *Lefty2* and *Otx2*, to assess how cells transitioned to EPL cells. These genes had similar expression patterns during the transition to EPL cells (Fig. 1F) but this behaviour changed under inhibitor-treated conditions (Fig. 5B).

Under standard culture conditions, L-proline-treated cells had significantly increased expression of *Dnmt3b* and *Otx2* between days 2 and 6 and *Fgf5* between days 4 and 6, but *Lefty2* expression was transiently increased at days 2 and 4 (Fig. 1Fii). Two inhibitors, SU5402 and LY294002, produced gene expression profiles that were less straightforward. Cells treated with SU5402 exhibited decreased *Dnmt3b* and *Fgf5* expression and increased *Lefty2* and *Otx2* expression, whereas cells treated with LY294002 had increased *Dnmt3b* expression but decreased *Lefty2* expression (Figs 4, 5).

These differences may reflect temporal expression patterns. Our control data showed transient increase in *Lefty2* expression on days 2 and 4 (Fig. 1Fii). By day 6, when we measured gene expression in inhibitor-treated cells, *Lefty2* expression had returned to baseline. This is in line with previous studies using EPL cells derived from embryoid bodies cultured in MEDII, which showed transient upregulation of *Lefty2* from days 1 to 4 (Harvey et al., 2010). This also explains the negative correlations between *Lefty2* and morphology changes (Fig. S6A). During the 6* *days of culture to EPL cells, we did not note any significant changes in expression of *Mixl1* (Figs 1Fii, 4G), indicating that cells remained within the pluripotency continuum and did not form mesendoderm.

To help assess this ambiguity between markers, we employed a functional assay which measured *T* expression as cells underwent spontaneous differentiation (Fig. S11A). EPL cells and some inhibitor-treated conditions (i.e. LY294002, low *Lefty2*, and SU5402, high *Lefty2*) tended to upregulate *T* fairly early (Fig. S11B), suggesting that the cells were further along the pluripotency continuum. ESCs and some inhibitor-treated conditions (i.e. PF-4708671 and rapamycin) took the longest to upregulate expression of *T*, suggesting that these were the most naïve cells. *Dmnt3b* and *Fgf5* expression at day 6 correlated highly with robust upregulation of *T* during the functional assay. No correlations were seen with *Lefty2*, suggesting that it is a poorer indicator of the position of a cell along the pluripotency continuum.

### Modelling reveals synergy and antagonism in emergent properties

Biological complexity was further highlighted when two or more inhibitors were used together. Two-way interaction effects were used to determine whether these pathways were independent (no interaction effects), antagonistic (where blocking two pathways simultaneously leads to a lower inhibition than the sum of the two inhibitors individually) or synergistic (where blocking two pathways simultaneously leads to higher inhibition than the sum of the two inhibitors individually). Only the emergent property MLR models were improved by including interaction effects (Table S2).

Antagonistic effects were seen for Mek1 and S6k, where the combination of inhibitors U0126 and PF-4708671 attenuated the inhibition of both cell number and proliferation compared with the use of each of the inhibitors alone (Figs 3Ciii-Diii, 5). The combination of mTor and S6k inhibitors (rapamycin and PF-4708671) attenuated the inhibition of cell number (Fig. 3Ciii) and the combination of inhibitors for Fgfr and mTor (SU5402 and rapamycin) promoted a change in colony morphology that was blocked by the individual inhibitors (Fig. 3Biii). These pathways likely coalesce on common downstream intermediates or transcription factors or suppress other pathways through crosstalk (Mendoza et al., 2011; Aksamitiene et al., 2012; Wang et al., 2013; Arkun, 2016).

Addition of both LY294002 and rapamycin resulted in strong synergistic effects that reduced cell numbers and proliferation and increased apoptosis (Figs 3Ciii-Eiii, 5), resulting in non-viable cells. Both pathways, when blocked individually, reduce proliferation and increase apoptosis (Fingar et al., 2002; Jirmanova et al., 2002; Murakami et al., 2004; Gross et al., 2005), and result in large defects in cell survival when blocked in combination in T cells, glioma cells and small cell lung cancer cells (Breslin et al., 2005; Takeuchi et al., 2005; Tsurutani et al., 2005).

### Understanding L-proline-mediated signalling in early embryogenesis

We have shown that L-proline activates multiple signalling pathways including the Mapk, Fgfr, Akt and mTor pathways in driving the transition of ESCs to EPL cells (Fig. 5). L-proline uptake through the Snat2 transporter likely directly alters cell signalling to modulate gene expression and emergent properties. It is also possible that other mechanisms such as metabolic flux and epigenetic changes alter the cellular landscape to facilitate autocrine cell signalling, activate signalling through crosstalk or change chromatin accessibility. This has been seen previously with autocrine Fgf4 activation of Fgfr as cells undergo lineage commitment (Kunath et al., 2007).

The L-proline-mediated transition of ESCs to EPL cells exemplifies the progression of cells from a naïve to primed or partially primed state in the pluripotency continuum (D'Aniello et al., 2017; Morgani et al., 2017; Cermola et al., 2021), and recapitulates aspects of peri- and post-implantation embryogenesis. These results are consistent with other growth factor-like roles for L-proline, including facilitating preimplantation embryo development (Morris et al., 2020; Treleaven et al., 2021) and steering differentiation of pluripotent cells towards neuroectoderm (Rathjen et al., 1999; 2002; Pelton et al., 2002; Harvey et al., 2010; Washington et al., 2010; Shparberg et al., 2019a,b). The L-proline-mediated transition along the pluripotency continuum provides a useful model to study embryonic development *in vitro*.

## MATERIALS AND METHODS

### Cell culture

All cell culture was performed at 37°C, 5% CO_2_ in a humidified incubator. D3 ESCs (Doetschman et al., 1985) were maintained in ESC self-renewal medium containing DMEM (Sigma-Aldrich), 10% FBS (AusGeneX), 1000 U/ml LIF (Neuromics), 0.1 mM β-Me (Sigma-Aldrich) and Pen/Strep (50 U/ml penicillin and 50 μg/ml streptomycin; both Sigma-Aldrich). Cells were grown as a monolayer, and passaged using Trypsin-EDTA (Sigma-Aldrich), and replated at 2000-20,000 live cells/cm^2^ (Glover et al., 2022).

ESCs were cultured to EPL cells by growing 20,000 cells/cm^2^ in EPL cell medium (90% DMEM, 10% FBS, Pen/Strep, 0.1 mM β-Me, 330 U/ml LIF, 400 μM L-proline; Sigma-Aldrich) for 6* *days, with passage every 2 days. As controls, ESCs were also cultured for 6* *days with 330 U/ml LIF or allowed to differentiate spontaneously without LIF or L-proline (Glover et al., 2022).

The effect of signalling pathway inhibitors (alone and in combination) on the transition of ESCs to EPL cells was assessed (Fig. 2D,E; Fig. S1D,E). The inhibitors were as follows: Mek1 inhibitor U0126 (U; 5 μM, Selleck); Fgfr inhibitor SU5402 (S; 5 μM, MedChem Express); Pi3k inhibitor LY294002 (L; 5 μM, Selleck); mTorc1 inhibitor rapamycin (R; 10 nM, Selleck) and S6k inhibitor PF-4708671 (P; 10 μM, MedChem Express). All inhibitors were dissolved in DMSO, and a vehicle control containing the maximum concentration (0.22%) of DMSO was included.

At days 2, 4 and 6, ESCs cultured in 1000 U/ml LIF or 330 U/ml LIF, and ESCs treated with L-proline±inhibitor(s) were analysed for three emergent properties (cell number, apoptosis and proliferation) and/or phosphorylation of various signalling pathway intermediates. At day 6, cells were also assessed for colony morphology, changes in gene expression and differentiation as embryoid bodies, as described below. Data were collected over five independent experiments.

### Measurement of cell number and colony morphology

Cell counts were measured with a haemocytometer following the addition of 0.4% Trypan Blue solution (Glover et al., 2022) to a single-cell suspension obtained following trypsinisation.

Colony morphology was quantified by images from an Olympus IX-81 inverted microscope. Images were deidentified and colony morphology scored based on a predetermined scale: round, domed (ESC) colonies were scored as 0; flat, irregular, partially transitioned colonies were scored as 1; and fully transitioned colonies consistent with EPL cells were scored as 2 (Glover et al., 2022). Scoring was performed on all colonies (10-40 per image) over three representative images from each condition. The sum of the score was divided by the total number of colonies scored, and then averaged across the three images to produce a final score.

### Analysis of differentiation potential using embryoid bodies

After 6* *days of culture in adherent culture, cells were passaged and 1.5×10^6^ were transferred to suspension culture plates and allowed to spontaneously differentiate without LIF or L-proline as EBs. EBs were collected at days 2, 3 and 4 and analysed by qRT-PCR for expression of the primitive streak marker *T* (primer sequences are provided in Table S3).

### Gene expression analysis using qRT-PCR

Total RNA was extracted from cells using GeneElute Mammalian Total RNA MiniPrep Kit (Sigma-Aldrich), including on-column DNase treatment to remove any contaminating DNA. RNA was converted to cDNA using a High-Capacity cDNA Reverse Transcriptase Kit (Applied Biosystems). qPCR was run on 10 μl reaction volumes containing 3 μl of 0.5 ng/μl cDNA, 2 μl of 1 μM primer (equal mix of forward and reverse primers; Table S3) and 5 μl of 2× SYBR Green master mix (Sigma-Aldrich) in a 384-well plate using a Roche LightCycler 480 with the following parameters: 15 min at 95°C, followed by 40 cycles of 30 s at 95°C, 60 s at 60°C, 30 s at 72°C. Thermal melt curves were obtained following this by increasing from 60°C to 95°C at 2.5°C/s. Threshold (C_t_) values were used to calculate relative expression to the reference gene *Actb* (encoding β-actin), employing REST v9 software. Results were normalised to untreated ESCs and transformed to log_2_ fold changes. All samples were tested to ensure that the C_t_ values for *Actb* were consistent (20±1 SD).

### Analysis of phosphorylation of signalling pathway intermediates

Cell samples were washed in ice-cold PBS and lysed (1 μl lysis buffer per 4×10^4^ cells) in the presence of protease and phosphatase inhibitors (Table S4). For data in Fig. 2C, cells were serum starved in 90% DMEM, 0.1% FBS and 0.1 mM β-Me for 4 h before sample collection. Cell lysates were incubated on ice for 10 min and then centrifuged at 4°C at 12,000 rpm (20,000 ***g***). The supernatant was loaded onto a 1.5 mm 12% polyacrylamide gel with a 4% stacking gel. Molecular weight markers (Bio-Rad Precision Plus Protein Standards) were also loaded. Electrophoresis was carried out in a Bio-Rad western blot chamber at 100 V for 2 h.

Following electrophoresis, proteins were transferred to a 0.45 μm nitrocellulose membrane (Bio-Rad) for 120 min at 100 V using a Bio-Rad transfer system. The membrane was blocked overnight in Odyssey Blocking buffer (LiCor) at 4°C, then washed 3×5 min in Tris buffered saline with Tween 20 (TBST) and then incubated with primary anti-phosphoprotein antibody overnight at 4°C with rocking. Anti-β-tubulin antibody was used to stain for the reference protein. The membranes were then washed 3×5 min in TBST before 2 h incubation at room temperature in the dark with fluorescently labelled secondary antibody. Primary and secondary antibodies were diluted in Odyssey Blocking buffer with 0.1% (v/v) Tween 20. For details of antibodies and dilutions, see Table S5.

Membranes were imaged using an Odyssey Infrared Imaging system (LiCor). Bands were identified based on the protein size, and the integrated intensity was calculated by Image Studio (LiCor). Selected bands for each antibody are shown in Fig. S1F. Background signal was accounted for by subtracting the integrated intensity of a size matched adjacent region (Fig. S1F). Data were normalised to β-tubulin to correct for differences in loading, and then to untreated ESCs.

### Apoptosis and proliferation analysis using flow cytometry

Flow cytometry was performed on a FACS Calibur and the results quantified using FlowJo software. Apoptosis was assayed using detection of annexin V. Live cells were centrifuged at 1200 rpm (2400 ***g***) for 2 min, washed in PBS and recentrifuged, and then resuspended in 100 μl annexin V binding buffer with either FITC-annexin V conjugated antibody (1:33 dilution in TBST) and Propidium Iodide staining solution (BD Pharmingen) or PE-annexin V conjugated antibody (1:33 dilution in TBST) and 7-AAD as per kit instructions (BD Pharmingen). Samples were analysed by flow cytometry within 30 min.

Proliferation was assayed using BrdU incorporation and processed using the FITC BrdU Flow Kit (BD Pharmingen). Briefly, BrdU was added to cells in culture at a final concentration of 10 μM and incubated for 1 h. Cells were passaged, washed in PBS, fixed in BD Cytofix/Cytoperm and stored at −80°C in BrdU freezing buffer until required. Thawed samples were then stained according to the manufacturer's instructions before flow cytometry.

### Statistical modelling and testing

We employed several methodologies to explore relationships within our data (correlation matrices and unsupervised learning) and to understand which signalling pathways contribute to changes in gene expression and emergent properties (supervised learning). Additional statistical analysis was performed, including one-way ANOVA with Tukey's or Dunnett's multiple comparisons test or a two-tailed *t*-test. Details for each test are included in the figure legends. All analyses were performed in R.

#### Correlation matrices

The correlation matrices were generated from paired data using Pearson correlations with significance levels based on rank correlation (*P<*0.05) (Hoyt et al., 2008). Matrices were calculated using the Hmisc R package, and parameters were ordered based on hierarchical clustering.

#### Unsupervised learning using kmeans

PCA was generated using the paired data matrix. Data were *z*-scored, and the silhouette score was calculated using the factoextra package. Kmeans clustering was performed using the stats package, using two centres, as indicated by the silhouette score, and 25 iterations. PCA was generated using the factoextra package, and samples were coloured by cluster (Fig. S7A). Boxplots were generated from the original paired data frame using the kmeans cluster assignments.

#### Supervised learning models

Gene expression and emergent properties (cell number, proliferation, apoptosis and morphology) were modelled with (1) standard MLR (Vittinghoff et al., 2012); (2) MLR with two-way interaction terms (Flanders et al., 1992); or (3) BRANNGP (Burden and Winkler, 2008; Winkler and Burden, 2012).

Before modelling, each inhibitor was encoded using a 1-hot descriptor (1 when present, 0 when absent). For modelling cell number, proliferation and apoptosis, 1-hot variables were also used to represent each experimental day (either 2, 4 or 6). Each condition with an average of three replicates was used as input for modelling, with replicates averaged before modelling. As conditions containing both LY294002 and rapamycin resulted in cells not being viable after day 2, these were excluded from modelling on days 4 and 6. Data were subject to Shapiro-Wilks test, and morphology, apoptosis and proliferation data were transformed to improve normality. To ensure linearity, the residuals for each model were also measured for normality using a Shapiro-Wilks test. Model fitting parameters, including adjusted R^2^ and σ values can be found in Table S1. Adjusted R^2^ was used for comparability across modelling styles. Models with a higher adjusted R^2^ and lower σ values are considered to have better fit. *F*-tests were also calculated to compare MLR with MLR with interaction effects (Table S2), where a *P*-value<0.05 indicates that the more complex models significantly improve the explanatory power of the model.

As this data contained all permutations and no predictive capacity was required, all the data were used to train models. To assess the range of responses from splitting the data, we generated 50 random 80% training/20% test models and profiled the range of responses seen in Table S6.

Models were generated using both the MLR and BRANNGP (sparse three-layer feedforward Bayesian regularised neural network with a Gaussian prior; Burden and Winkler, 2008). These were implemented in the in house software package Biomodeller. The latter method automatically optimises the complexity of the model (number of weights) to maximise predictivity. Models were trained until they reached the maximum of the evidence for the model so no validation set was required to provide a stopping criterion, which is important given the small dataset sizes. These models employed two neurons in the hidden layer, linear transfer functions in the input and output layer neurons and sigmoidal transfer functions in the hidden layer neurons. Data applied to the input layer was column scaled. See Burden and Winkler (1999) for a detailed explanation of BRANNGP methodology.

## Supplementary Material

10.1242/develop.201704_sup1 Supplementary information

## References

[DEV201704C1] Acampora, D., Di Giovannantonio, L. G. and Simeone, A. (2013). Otx2 is an intrinsic determinant of the embryonic stem cell state and is required for transition to a stable epiblast stem cell condition. *Development* 140, 43-55. 10.1242/dev.085290 23154415

[DEV201704C2] Aguilar, J. and Reyley, M. (2005). The uterine tubal fluid: secretion, composition and biological effects. *Anim. Reprod. Sci.* 2, 91-105.

[DEV201704C3] Aksamitiene, E., Kiyatkin, A. and Kholodenko, B. N. (2012). Cross-talk between mitogenic Ras/MAPK and survival PI3K/Akt pathways: a fine balance. *Biochem. Soc. Trans.* 40, 139-146. 10.1042/BST20110609 22260680

[DEV201704C4] Arkun, Y. (2016). Dynamic modeling and analysis of the cross- talk between insulin/AKT and MAPK/ERK signaling pathways. *PLoS One* 11, e0149684. 10.1371/journal.pone.0149684 26930065 PMC4773096

[DEV201704C5] Audet, J. (2010). Adventures in time and space: nonlinearity and complexity of cytokine effects on stem cell fate decisions. *Biotechnol. Bioeng.* 106, 173-182. 10.1002/bit.22708 20198618

[DEV201704C6] Bahrami, M., Morris, M. B. and Day, M. L. (2019). Amino acid supplementation of a simple inorganic salt solution supports efficient in vitro maturation (IVM) of bovine oocytes. *Sci. Rep.* 9, 11739. 10.1038/s41598-019-48038-y 31409817 PMC6692353

[DEV201704C7] Bazer, F. W., Johnson, G. A. and Wu, G. (2015). Amino acids and conceptus development during the peri-implantation period of pregnancy. *Adv. Exp. Med. Biol.* 843, 23-52. 10.1007/978-1-4939-2480-6_2 25956294

[DEV201704C8] Brennan, J., Lu, C. C., Norris, D. P., Rodriguez, T. A., Beddington, R. S. and Robertson, E. J. (2001). Nodal signalling in the epiblast patterns the early mouse embryo. *Nature* 411, 965-969. 10.1038/35082103 11418863

[DEV201704C9] Breslin, E. M., White, P. C., Shore, A. M., Clement, M. and Brennan, P. (2005). LY294002 and rapamycin co-operate to inhibit T-cell proliferation. *Br. J. Pharmacol.* 144, 791-800. 10.1038/sj.bjp.0706061 15778701 PMC1576062

[DEV201704C10] Burden, F. R. and Winkler, D. A. (1999). Robust QSAR models using bayesian regularized neural networks. *J. Med. Chem.* 42, 3183-3187. 10.1021/jm980697n 10447964

[DEV201704C11] Burden, F. and Winkler, D. (2008). Bayesian regularization of neural networks. *Methods Mol. Biol.* 458, 22-44. 10.1007/978-1-60327-101-1_3 19065804

[DEV201704C12] Casalino, L., Comes, S., Lambazzi, G., De Stefano, B., Filosa, S., De Falco, S., De Cesare, D., Minchiotti, G. and Patriarca, E. J. (2011). Control of embryonic stem cell metastability by L-proline catabolism. *J. Mol. Cell Biol.* 3, 108-122. 10.1093/jmcb/mjr001 21307025

[DEV201704C13] Cermola, F., D'Aniello, C., Tatè, R., De Cesare, D., Martinez-Arias, A., Minchiotti, G. and Patriarca, E. J. (2021). Gastruloid development competence discriminates different states of pluripotency. *Stem Cell Rep.* 16, 354-369. 10.1016/j.stemcr.2020.12.013 PMC7878839 33482102

[DEV201704C14] Cetin, I., de Santis, M. S. N., Taricco, E., Radaelli, T., Teng, C., Ronzoni, S., Spada, E., Milani, S. and Pardi, G. (2005). Maternal and fetal amino acid concentrations in normal pregnancies and in pregnancies with gestational diabetes mellitus. *Am. J. Obstet. Gynecol.* 192, 610-617. 10.1016/j.ajog.2004.08.011 15696011

[DEV201704C15] Chang, K. H. and Zandstra, P. W. (2004). Quantitative screening of embryonic stem cell differentiation: endoderm formation as a model. *Biotechnol. Bioeng.* 88, 287-298. 10.1002/bit.20242 15486933

[DEV201704C16] Chang, F., Lee, J. T., Navolanic, P. M., Steelman, L. S., Shelton, J. G., Blalock, W. L., Franklin, R. A. and Mccubrey, J. A. (2003). Involvement of PI3K/Akt pathway in cell cycle progression, apoptosis, and neoplastic transformation: a target for cancer chemotherapy. *Leukemia* 17, 590-603. 10.1038/sj.leu.2402824 12646949

[DEV201704C17] Chen, H., Guo, R., Zhang, Q., Guo, H., Yang, M., Wu, Z., Gao, S., Liu, L. and Chen, L. (2015). Erk signaling is indispensable for genomic stability and self-renewal of mouse embryonic stem cells. *Proc. Natl. Acad. Sci. USA* 112, E5936-E5943. 10.1073/pnas.1516319112 26483458 PMC4640739

[DEV201704C18] Cherepkova, M. Y., Sineva, G. S. and Pospelov, V. A. (2016). Leukemia inhibitory factor (LIF) withdrawal activates mTOR signaling pathway in mouse embryonic stem cells through the MEK/ERK/TSC2 pathway. *Cell Death Dis.* 7, e2050. 10.1038/cddis.2015.387 26775702 PMC4816172

[DEV201704C19] Comes, S., Gagliardi, M., Laprano, N., Fico, A., Cimmino, A., Palamidessi, A., De Cesare, D., De Falco, S., Angelini, C., Scita, G. et al. (2013). L-proline induces a mesenchymal-like invasive program in embryonic stem cells by remodeling H3K9 and H3K36 methylation. *Stem Cell Rep.* 1, 307-321. 10.1016/j.stemcr.2013.09.001 PMC3849245 24319666

[DEV201704C20] Coucouvanis, E. and Martin, G. R. (1999). BMP signaling plays a role in visceral endoderm differentiation and cavitation in the early mouse embryo. *Development* 126, 535-546. 10.1242/dev.126.3.535 9876182

[DEV201704C21] Dailey, L., Ambrosetti, D., Mansukhani, A. and Basilico, C. (2005). Mechanisms underlying differential responses to FGF signaling. *Cytokine Growth Factor Rev.* 16, 233-247. 10.1016/j.cytogfr.2005.01.007 15863038

[DEV201704C22] D'Aniello, C., Fico, A., Casalino, L., Guardiola, O., Di Napoli, G., Cermola, F., De Cesare, D., Tatã¨, R., Cobellis, G., Patriarca, E. J. et al. (2015). A novel autoregulatory loop between the Gcn2-Atf4 pathway and L-Proline metabolism controls stem cell identity. *Cell Death Differ.* 22, 1094-1105. 10.1038/cdd.2015.24 25857264 PMC4572871

[DEV201704C23] D'Aniello, C., Habibi, E., Cermola, F., Paris, D., Russo, F., Fiorenzano, A., Di Napoli, G., Melck, D. J., Cobellis, G., Angelini, C. et al. (2017). Vitamin C and L-Proline antagonistic effects capture alternative states in the pluripotency continuum. *Stem Cell Rep.* 8, 1-10. 10.1016/j.stemcr.2016.11.011 PMC5233408 28017658

[DEV201704C24] Doetschman, T. C., Eistetter, H., Katz, M., Schmidt, W. and Kemler, R. (1985). The *in vitro* development of blastocyst-derived embryonic stem cell lines: formation of visceral yolk sac, blood islands and myocardium. *J. Embryol. Exp. Morphol.* 87, 27-45. 3897439

[DEV201704C25] Dowling, R. J. O., Horiuchi, K. Y., Manos, E. J., Daulerio, A. J., Stradley, D. A., Feeser, W. S., Van Dyk, D. E., Pitts, W. J., Earl, R. A., Hobbs, F. et al. (2010). mTORC1-mediated cell proliferation, but not cell growth, controlled by the 4E-BPs. *Science* 328, 1172. 10.1126/SCIENCE.1187532 20508131 PMC2893390

[DEV201704C26] Epa, V. C., Yang, J., Mei, Y., Hook, A. L., Langer, R., Anderson, D. G., Davies, M. C., Alexander, M. R. and Winkler, D. A. (2013). Europe PMC Funders Group Modelling human embryoid body cell adhesion to a combinatorial library of polymer surfaces. *J. Mater. Chem.* 22, 20902-20906. 10.1039/C2JM34782B.Modelling PMC3787298 24092955

[DEV201704C27] Favata, M. F., Horiuchi, K. Y., Manos, E. J., Daulerio, A. J., Stradley, D. A., Feeser, W. S., Van Dyk, D. E., Pitts, W. J., Earl, R. A., Hobbs, F. et al. (1998). Identification of a novel inhibitor of mitogen-activated protein kinase. *J. Biol. Chem.* 273, 18623-18632. 10.1074/jbc.273.29.18623 9660836

[DEV201704C28] Fingar, D. C., Salama, S., Tsou, C., Harlow, E. and Blenis, J. (2002). Mammalian cell size is controlled by mTOR and its downstream targets S6K1 and 4EBP1/eIF4E. *Genes Dev.* 16, 1472-1487. 10.1101/gad.995802 12080086 PMC186342

[DEV201704C29] Flanders, W. D., Dersimonian, R. and Freedman, D. S. (1992). Interpretation of linear regression models that include transformations or interaction terms. *Ann. Epidemiol.* 2, 735-744. 10.1016/1047-2797(92)90018-L 1342325

[DEV201704C30] Gardner, D. K. and Lane, M. (1993). Amino acids and ammonium regulate mouse embryo development in culture. *Biol. Reprod.* 48, 377-385. 10.1095/biolreprod48.2.377 8439627

[DEV201704C31] Glover, H. J., Shparberg, R. A. and Morris, M. B. (2022). L-proline supplementation drives self-renewing mouse embryonic stem cells to a partially primed pluripotent state: the early primitive ectoderm-like cell. *Methods Mol. Biol.* 2490, 11-24. 10.1007/978-1-0716-2281-0_2 35486235

[DEV201704C32] Gross, V. S., Hess, M. and Cooper, G. M. (2005). Mouse embryonic stem cells and preimplantation embryos require signaling through the phosphatidylinositol 3-kinase pathway to suppress apoptosis. *Mol. Reprod. Dev.* 70, 324-332. 10.1002/mrd.20212 15625701

[DEV201704C33] Harris, S. E., Gopichandran, N., Picton, H. M., Leese, H. J. and Orsi, N. M. (2005). Nutrient concentrations in murine follicular fluid and the female reproductive tract. *Theriogenology* 64, 992-1006. 10.1016/j.theriogenology.2005.01.004 16054501

[DEV201704C34] Hart, A. H., Hartley, L., Sourris, K., Stadler, E. S., Li, R., Stanley, E. G., Tam, P. P. L., Elefanty, A. G. and Robb, L. (2002). Mixl1 is required for axial mesendoderm morphogenesis and patterning in the murine embryo. *Development* 129, 3597-3608. 10.1242/dev.129.15.3597 12117810

[DEV201704C35] Harvey, N. T., Hughes, J. N., Lonic, A., Yap, C., Long, C., Rathjen, P. D. and Rathjen, J. (2010). Response to BMP4 signalling during ES cell differentiation defines intermediates of the ectoderm lineage. *J. Cell Sci.* 123, 1796-1804. 10.1242/jcs.047530 20427322

[DEV201704C36] Hoogland, S. H. A. and Marks, H. (2021). Developments in pluripotency: a new formative state. *Cell Res.* 31, 493-494. 10.1038/s41422-021-00494-w 33731854 PMC8089087

[DEV201704C37] Hoyt, W. T., Imel, Z. E. and Chan, F. (2008). Multiple regression and correlation techniques: recent controversies and best practices. *Rehabil. Psychol.* 53, 321-339. 10.1037/a0013021

[DEV201704C38] Huang, G., Yan, H., Ye, S., Tong, C. and Ying, Q. L. (2014). STAT3 phosphorylation at tyrosine 705 and serine 727 differentially regulates mouse ESC fates. *Stem Cells* 32, 1149. 10.1002/STEM.1609 24302476 PMC4181708

[DEV201704C39] Ireland, R. G., Kibschull, M., Audet, J., Ezzo, M., Hinz, B., Lye, S. J. and Simmons, C. A. (2020). Combinatorial extracellular matrix microarray identifies novel bioengineered substrates for xeno-free culture of human pluripotent stem cells. *Biomaterials* 248, 120017. 10.1016/j.biomaterials.2020.120017 32283392

[DEV201704C40] Jakobsen, R. B., Østrup, E., Zhang, X., Mikkelsen, T. S. and Brinchmann, J. E. (2014). Analysis of the effects of five factors relevant to in vitro chondrogenesis of human mesenchymal stem cells using factorial design and high throughput mRNA-profiling. *PLoS One* 9, e96615. 10.1371/journal.pone.0096615 24816923 PMC4015996

[DEV201704C41] Jirmanova, L., Afanassieff, M., Gobert-Gosse, S., Markossian, S. and Savatier, P. (2002). Differential contributions of ERK and PI3-kinase to the regulation of cyclin D1 expression and to the control of the G1/S transition in mouse embryonic stem cells. *Oncogene* 21, 5515-5528. 10.1038/sj.onc.1205728 12165850

[DEV201704C42] Julkunen, H., Chu, J., Shen, X., Wang, J. and Orkin, S. H. (2020). Leveraging multi-way interactions for systematic prediction of pre-clinical drug combination effects. *Nat. Commun.* 11, 6136. 10.1038/s41467-020-19950-z 33262326 PMC7708835

[DEV201704C43] Kim, J., Chu, J., Shen, X., Wang, J. and Orkin, S. H. (2008). An extended transcriptional network for pluripotency of embryonic stem cells. *Cell* 132, 1049-1061. 10.1016/j.cell.2008.02.039 18358816 PMC3837340

[DEV201704C44] Kunath, T., Saba-El-Leil, M. K., Almousailleakh, M., Wray, J., Meloche, S. and Smith, A. (2007). FGF stimulation of the Erk1/2 signalling cascade triggers transition of pluripotent embryonic stem cells from self-renewal to lineage commitment. *Development* 134, 2895-2902. 10.1242/dev.02880 17660198

[DEV201704C45] Lane, M. and Gardner, D. K. (1997). Nonessential amino acids and glutamine decrease the time of the first three cleavage divisions and increase compaction of mouse zygotes in vitro. *J. Assist. Reprod. Genet.* 14, 398-403. 10.1007/BF02766148 9285325 PMC3454776

[DEV201704C46] Lanner, F. and Rossant, J. (2010). The role of FGF/Erk signaling in pluripotent cells. *Development* 137, 3351-3360. 10.1242/dev.050146 20876656

[DEV201704C47] Li, Q., Xing, X. B., Chen, T. T., Li, R. X., Dai, J., Sheng Q. H., Xin, S. M., Zhu, L. L., Jin, Y., Pei, G. et al. (2011). Large scale phosphoproteome profiles comprehensive features of mouse embryonic stem cells. *Mol. Cell. Proteomics* 10, M110.001750. 10.1074/mcp.M110.001750 PMC3069338 21149613

[DEV201704C48] Lonic, A. (2006). *Molecular mechanism of L-proline induced EPL-cell formation*. University of Adelaide.

[DEV201704C49] Mendoza, M. C., Er, E. E. and Blenis, J. (2011). The Ras-ERK and PI3K-mTOR pathways: cross-talk and compensation. *Trends Biochem. Sci.* 36, 320-328. 10.1016/j.tibs.2011.03.006 21531565 PMC3112285

[DEV201704C50] Minchiotti, G., D'Aniello, C., Fico, A., De Cesare, D. and Patriarca, E. J. (2022). Capturing transitional pluripotency through proline metabolism. *Cells* 11, 2125. 10.3390/CELLS11142125 35883568 PMC9323356

[DEV201704C51] Mohammadi, M., McMahon, G., Sun, L., Tang, C., Hirth, P., Yeh, B. K., Hubbard, S. R. and Schlessinger, J. (1997). Structures of the tyrosine kinase domain of fibroblast growth factor receptor in complex with inhibitors. *Science* 276, 955-960. 10.1126/science.276.5314.955 9139660

[DEV201704C52] Morgani, S., Nichols, J. and Hadjantonakis, A. K. (2017). The many faces of Pluripotency: In vitro adaptations of a continuum of in vivo states. *BMC Dev. Biol.* 17, 10-12. 10.1186/s12861-017-0150-4 28610558 PMC5470286

[DEV201704C53] Morris, M. B., Ozsoy, S., Zada, M., Zada, M., Zamfirescu, R. C., Todorova, M. G. and Day, M. L. (2020). Selected amino acids promote mouse pre-implantation embryo development in a growth factor-like manner. *Front. Physiol.* 11, 140. 10.3389/fphys.2020.00140 32210831 PMC7076138

[DEV201704C54] Murakami, M., Ichisaka, T., Maeda, M., Oshiro, N., Hara, K., Edenhofer, F., Kiyama, H., Yonezawa, K. and Yamanaka, S. (2004). mTOR is essential for growth and proliferation in early mouse embryos and embryonic stem cells. *Mol. Cell. Biol.* 24, 6710-6718. 10.1128/MCB.24.15.6710-6718.2004 15254238 PMC444840

[DEV201704C55] Nawroth, R., Stellwagen, F., Schulz, W. A., Stoehr, R., Hartmann, A., Krause, B. J., Gschwend, J. E. and Retz, M. (2011). S6K1 and 4E-BP1 are independent regulated and control cellular growth in bladder cancer. *PLoS One* 6, e27509. 10.1371/JOURNAL.PONE.0027509 22110663 PMC3216974

[DEV201704C56] Panina, S. B., Pei, J., Baran, N., Konopleva, M. and Kirienko, N. V. (2020). Utilizing synergistic potential of mitochondria-targeting drugs for leukemia therapy. *Front. Oncol.* 10, 435. 10.3389/fonc.2020.00435 32318340 PMC7146088

[DEV201704C57] Patriarca, E. J., Cermola, F., D'Aniello, C., Fico, A., Guardiola, O., De Cesare, D. and Minchiotti, G. (2021). The multifaceted roles of proline in cell behavior. *Front. Cell Dev. Biol.* 9, 728576. 10.3389/FCELL.2021.728576 34458276 PMC8397452

[DEV201704C58] Pearce, L. R., Alton, G. R., Richter, D. T., Kath, J. C., Lingardo, L., Chapman, J., Hwang, C. and Alessi, D. R. (2010). Characterization of PF-4708671, a novel and highly specific inhibitor of p70 ribosomal S6 kinase (S6K1). *Biochem. J.* 431, 245-255. 10.1042/BJ20101024 20704563

[DEV201704C59] Pelton, T. A., Sharma, S., Schulz, T. C., Rathjen, J. and Rathjen, P. D. (2002). Transient pluripotent cell populations during primitive ectoderm formation: correlation of *in vivo* and *in vitro* pluripotent cell development. *J. Cell Sci.* 115, 329-339. 10.1242/jcs.115.2.329 11839785

[DEV201704C60] Pons, B., Peg, V., Vázquez-Sánchez, M. A., López-Vicente, L., Argelaguet, E., Coch, L., Martínez, A., Hernández-Losa, J., Armengol, G., Ramon, Y. et al. (2011). The effect of p-4E-BP1 and p-eIF4E on cell proliferation in a breast cancer model. *Int. J. Oncol.* 39, 1337-1345. 10.3892/IJO.2011.1118 21750861

[DEV201704C61] Prudhomme, W. A., Duggar, K. H. and Lauffenburger, D. A. (2004). Cell population dynamics model for deconvolution of murine embryonic stem cell self-renewal and differentiation responses to cytokines and extracellular matrix. *Biotechnol. Bioeng.* 88, 264-272. 10.1002/bit.20244 15486930

[DEV201704C62] Rathjen, J., Lake, J.-A., Bettess, M. D., Washington, J. M., Chapman, G. and Rathjen, P. D. (1999). Formation of a primitive ectoderm like cell population, EPL cells, from ES cells in response to biologically derived factors. *J. Cell Sci.* 112, 601-612. 10.1242/jcs.112.5.601 9973595

[DEV201704C63] Rathjen, J., Haines, B. P., Hudson, K. M., Nesci, A., Dunn, S. and Rathjen, P. D. (2002). Directed differentiation of pluripotent cells to neural lineages: homogeneous formation and differentiation of a neurectoderm population. *Development* 129, 2649-2661. 10.1242/dev.129.11.2649 12015293

[DEV201704C64] Rengasamy, D., Mase, J. M., Kumar, A., Rothwell, B., Torres, M. T., Alexander, M. R., Winkler, D. A. and Figueredo, G. P. (2022). Feature importance in machine learning models: a fuzzy information fusion approach. *Neurocomput.* 511, 163-174. 10.1016/j.neucom.2022.09.053

[DEV201704C65] Rivera-Pérez, J. A. and Hadjantonakis, A. K. (2015). The dynamics of morphogenesis in the early mouse embryo. *Cold Spring Harbor Perspect. Biol.* 7, a015867. 10.1101/CSHPERSPECT.A015867 PMC4277506 24968703

[DEV201704C66] Sabers, C. J., Martin, M. M., Brunn, G. J., Williams, J. M., Dumont, F. J., Wiederrecht, G. and Abraham, R. T. (1995). Isolation of a protein target of the FKBP12-rapamycin complex in mammalian cells. *J. Biol. Chem.* 270, 815-822. 10.1074/jbc.270.2.815 7822316

[DEV201704C67] Shparberg, R. A., Glover, H. J. and Morris, M. B. (2019a). Embryoid body differentiation of mouse embryonic stem cells into neurectoderm and neural progenitors. *Methods Mol. Biol.* 2029, 273-285. 10.1007/978-1-4939-9631-5_21 31273749

[DEV201704C68] Shparberg, R., Glover, H. and Morris, M. B. (2019b). Modeling mammalian commitment to the neural lineage using embryos and embryonic stem cells. *Front. Physiol.* 10, 705. 10.3389/FPHYS.2019.00705 31354503 PMC6637848

[DEV201704C69] Smith, A. (2017). Formative pluripotency: the executive phase in a developmental continuum. *Development* 144, 365-373. 10.1242/dev.142679 28143843 PMC5430734

[DEV201704C70] Snow, M. (1977). Gastrulation in the mouse: growth and regionalization of the epiblast. *J. Embryol. Exp. Morph.* 42, 293-303. 10.1242/dev.42.1.293

[DEV201704C71] Sorokin, M., Kholodenko, R., Suntsova, M., Malakhova, G., Garazha, A., Kholodenko, I., Poddubskaya, E., Lantsov, D., Stilidi, I., Arhiri, P. et al. (2018). Oncobox bioinformatical platform for selecting potentially effective combinations of target cancer drugs using high-throughput gene expression data. *Cancers* 10, 365. 10.3390/cancers10100365 30274248 PMC6209915

[DEV201704C72] Stavridis, M. P., Collins, B. J. and Storey, K. G. (2010). Retinoic acid orchestrates fibroblast growth factor signalling to drive embryonic stem cell differentiation. *Development* 137, 881-890. 10.1242/dev.043117 20179094 PMC2834455

[DEV201704C73] Stead, E., White, J., Faast, R., Conn, S., Goldstone, S., Rathjen, J., Dhingra, U., Rathjen, P., Walker, D. and Dalton, S. (2002). Pluripotent cell division cycles are driven by ectopic Cdk2, cyclin A/E and E2F activities. *Oncogene* 21, 8320-8333. 10.1038/sj.onc.1206015 12447695

[DEV201704C74] Takahashi, K., Murakami, M. and Yamanaka, S. (2005). Role of the phosphoinositide 3-kinase pathway in mouse embryonic stem (ES) cells. *Biochem. Soc. Trans.* 33, 1522-1525. 10.1042/BST0331522 16246160

[DEV201704C75] Takeuchi, H., Kondo, Y., Fujiwara, K., Kanzawa, T., Aoki, H., Mills, G. B. and Kondo, S. (2005). Synergistic augmentation of rapamycin-induced autophagy in malignant glioma cells by phosphatidylinositol 3-kinase/protein kinase B inhibitors. *Cancer Res.* 65, 3336-3346. 10.1158/0008-5472.CAN-04-3640 15833867

[DEV201704C76] Tan, B. S. N., Lonic, A., Morris, M. B., Rathjen, P. D. and Rathjen, J. (2011). The amino acid transporter SNAT2 mediates l-proline-induced differentiation of ES cells. *Am. J. Physiol. Cell Physiol.* 300, C1270-C1279. 10.1152/ajpcell.00235.2010 21346154

[DEV201704C77] Tan, B. S. N., Kwek, J., Wong, C. K., Saner, N. J., Yap, C., Felquer, F., Morris, M. B., Gardner, D. K., Rathjen, P. D. and Rathjen, J. (2016). Src family kinases and p38 Mitogen-Activated Protein Kinases regulate pluripotent cell differentiation in culture. *PLoS One*. 11, e0163244. 10.1371/journal.pone.0163244 27723793 PMC5056717

[DEV201704C78] Treleaven, T., Hardy, M. L. M., Guttman-Jones, M., Morris, M. B. and Day, M. L. (2021). In vitro fertilisation of mouse oocytes in l-proline and l-pipecolic acid improves subsequent development. *Cells* 10, 1352. 10.3390/cells10061352 34072568 PMC8229504

[DEV201704C79] Tsurutani, J., West, K. A., Sayyah, J., Gills, J. J. and Dennis, P. A. (2005). Inhibition of the phosphatidylinositol 3-kinase/Akt/mammalian target of rapamycin pathway but not the MEK/ERK pathway attenuates laminin-mediated small cell lung cancer cellular survival and resistance to imatinib mesylate or chemotherapy. *Cancer Res.* 65, 8423-8432. 10.1158/0008-5472.CAN-05-0058 16166321

[DEV201704C80] Vittinghoff, E., Glidden, D. V., Shiboski, S. C. and McCulloch, C. E. (2012). *Regression Methods in Biostatistics*. Springer New York.

[DEV201704C81] Vlahos, C. J., Matter, W. F., Hui, K. Y. and Brown, R. F. (1994). A specific inhibitor of phosphatidylinositol 3-kinase, 2-(4-morpholinyl)-8-phenyl-4H-1-benzopyran-4-one (LY294002). *J. Biol. Chem.* 269, 5241-5248. 10.1016/S0021-9258(17)37680-9 8106507

[DEV201704C82] Wang, C., Cigliano, A., Delogu, S., Armbruster, J., Dombrowski, F., Evert, M., Chen, X. and Calvisi, D. (2013). Functional crosstalk between AKT/mTOR and Ras/MAPK pathways in hepatocarcinogenesis: implications for the treatment of human liver cancer. *Cell Cycle* 12, 1999-2010. 10.4161/cc.25099 23759595 PMC3737302

[DEV201704C83] Wang, X., Xiang, Y., Yu, Y., Wang, R., Zhang, Y., Xu, Q., Sun, H., Zhao, Z.-A., Jiang, X., Wang, X. et al. (2021). Formative pluripotent stem cells show features of epiblast cells poised for gastrulation. *Cell Res.* 31, 526-541. 10.1038/s41422-021-00477-x 33608671 PMC8089102

[DEV201704C84] Washington, J. M., Rathjen, J., Felquer, F., Lonic, A., Bettess, M. D., Hamra, N., Semendric, L., Tan, B. S. N., Lake, J.-A., Keough, R. A. et al. (2010). L-Proline induces differentiation of ES cells: a novel role for an amino acid in the regulation of pluripotent cells in culture. *Am. J. Physiol. Cell Physiol.* 298, 982-992. 10.1152/ajpcell.00498.2009 20164384

[DEV201704C85] Watanabe, D., Suetake, I., Tada, T. and Tajima, S. (2002). Stage- and cell-specific expression of Dnmt3a and Dnmt3b during embryogenesis. *Mech. Dev.* 118, 187-190. 10.1016/S0925-4773(02)00242-3 12351185

[DEV201704C86] Van Winkle, L. J. (2001). Amino acid transport regulation and early embryo development. *Biol. Reprod.* 64, 1-12. 10.1095/biolreprod64.1.1 11133652

[DEV201704C87] Van Winkle, L. J., Tesch, J. K., Shah, A. and Campione, A. L. (2006). System B0,+ amino acid transport regulates the penetration stage of blastocyst implantation with possible long-term developmental consequences through adulthood. *Hum. Reprod. Update* 12, 145-157. 10.1093/humupd/dmi044 16251251

[DEV201704C88] Winkler, D. A. and Burden, F. R. (2012). Robust, quantitative tools for modelling *ex-vivo* expansion of haematopoietic stem cells and progenitors. *Mol. Biosyst.* 8, 913-920. 10.1039/c2mb05439f 22282302

[DEV201704C89] Woolf, P. J., Prudhomme, W., Daheron, L., Daley, G. Q. and Lauffenburger, D. A. (2005). Bayesian analysis of signaling networks governing embryonic stem cell fate decisions. *Bioinformatics* 21, 741-753. 10.1093/bioinformatics/bti056 15479714

[DEV201704C90] Yellen, P., Saqcena, M., Salloum, D., Feng, J., Preda, A., Xu, L., Rodrik-Outmezguine, V. and Foster, D. A. (2011). High-dose rapamycin induces apoptosis in human cancer cells by dissociating mTOR complex 1 and suppressing phosphorylation of 4E-BP1. *Cell Cycle* 10, 3948-3956. 10.4161/CC.10.22.18124 22071574 PMC3266120

[DEV201704C91] Yu, J. S. L. and Cui, W. (2016). Proliferation, survival and metabolism: the role of PI3K/AKT/ mTOR signalling in pluripotency and cell fate determination. *Development* 143, 3050-3060. 10.1242/dev.137075 27578176

