# Supplementary material for: Signalling pathway crosstalk stimulated by L-proline drives mouse embryonic stem cells to primitive-ectoderm-like cells
Source: Development. 2023 Oct 26;150(20):dev201704. doi: 10.1242/dev.201704 (PMC10652046; doi:10.1242/dev.201704)
Supplement: Supplementary information [file develop-150-201704-s1.pdf]

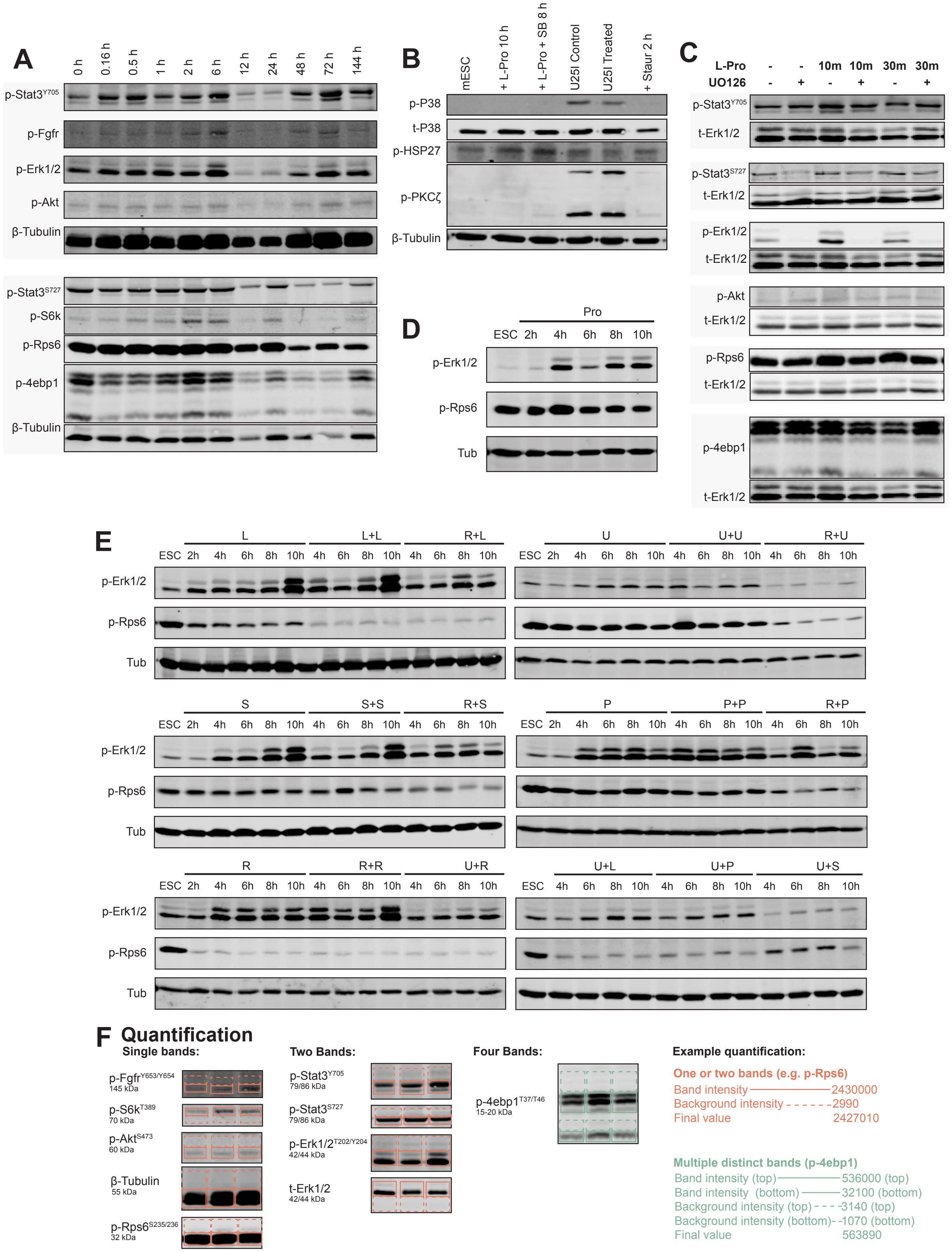

Development • Supplementary information

Fig. S1. Representative western blots show changes in pathway phosphorylation. A. Naïve ESCs were grown in medium containing 330 U/mL LIF + 400 µM L-proline for up to 6 days (144 h). Cell lysates were analysed by western blotting for: p-Stat3^{Y705} or p-Stat3^{S727}, p-Fgfr$^{Y653/Y654}$, p-Erk1/2$^{T202/Y204}$, p-Akt^{S473}, p-S6k^{T389}, p-Rps6$^{S235/S236}$ and p-4ebp1$^{T37/T46}$ and β-Tubulin as a loading control. **B.** Naïve ESCs were treated with either 400 µM L-proline, the P38 inhibitor SB205580 (SB, 10 µM), or staurosporine (1 mM) for the times indicated. As a positive control for P38 phosphorylation, protein lysates from the U25I human glioblastoma cell line were also used. Cell lysates were taken and analysed using western blotting for p-P38$^{T180/Y182}$, total P38 (t-P38), p-Hsp27$^{S78/S82}$, p-Pkcζ^{T410} and β-Tubulin as a loading control. **C.** Naïve ESCs were serum starved in 0.1% FBS for 4 h, with 5 µM U0126 added for the final 30 min. Cells were then left untreated or treated with 1 mM L-proline for 10 or 30 min. Cell lysates were analysed by western blotting for: p-Stat3^{Y705} or p-Stat3^{S727}, p-Fgfr$^{Y653/Y654}$, p-Erk1/2$^{T202/Y204}$, p-Akt^{S473}, p-Rps6$^{S235/S236}$ and p-4ebp1$^{T37/T46}$ and total Erk1/2 as a loading control. **D.** 400 µM L-proline and signalling pathway inhibitors were added to naïve ESCs growing in 1000 U/mL LIF at 0 h. After 2 h, cell samples were analysed for p-Erk1/2$^{T202/Y204}$and p-Rps6$^{S235/S236}$ and β-Tubulin as a loading control. **E.** 400 µM L-proline and signalling pathway inhibitors (1i) were added to naïve ESCs growing in 1000 U/mL LIF at 0 h. At 2, 4, 6, or 8 h, a second dose of the same inhibitor or a different inhibitor (2i) was added, and samples were collected 2 h later Cell samples were analysed for p-Erk1/2$^{T202/Y204}$and p-Rps6$^{S235/S236}$ and β-Tubulin as a loading control. **F.** Western blot quantification in Fig. 2 was calculated by measuring the integrated intensity of the band or bands with Image Studio. The integrated intensity of an adjacent paired region was subtracted to account for background signal. Worked examples are shown for proteins with a one or two bands (e.g., p-Rps6) and multiple bands (p-4ebp1).

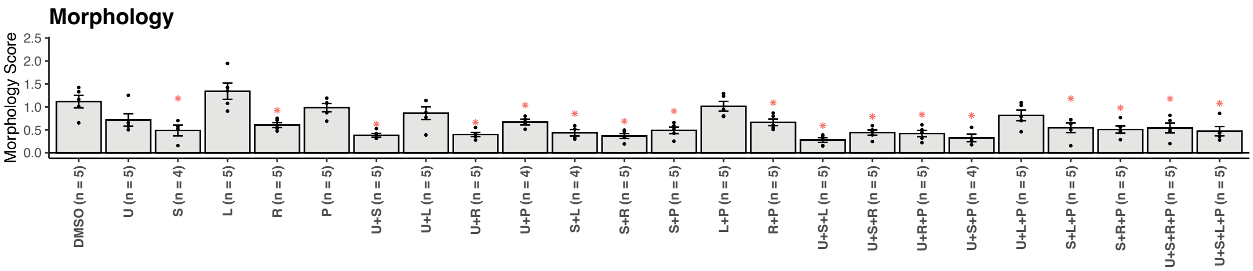

Fig. S2. Morphology data by inhibitor combination. Naïve ESCs were cultured over 6 days in 330 U/mL LIF + 400 µM L-proline with combinations of five inhibitors (U: U0126; S: SU5402; L: LY294002; R: rapamycin; P: PF-4708671). Morphology scoring was performed on day 6. Conditions containing L+R (navy) were considered non-viable at day 2 and no data is available for this combination. Data is shown as mean and SEM with individual data points. Data were analysed using one-way ANOVA with Dunnett's multiple comparisons test to cells grown in 330 U/mL LIF + 400 µM L-proline + DMSO, *$P < 0.05$.

Development • Supplementary information

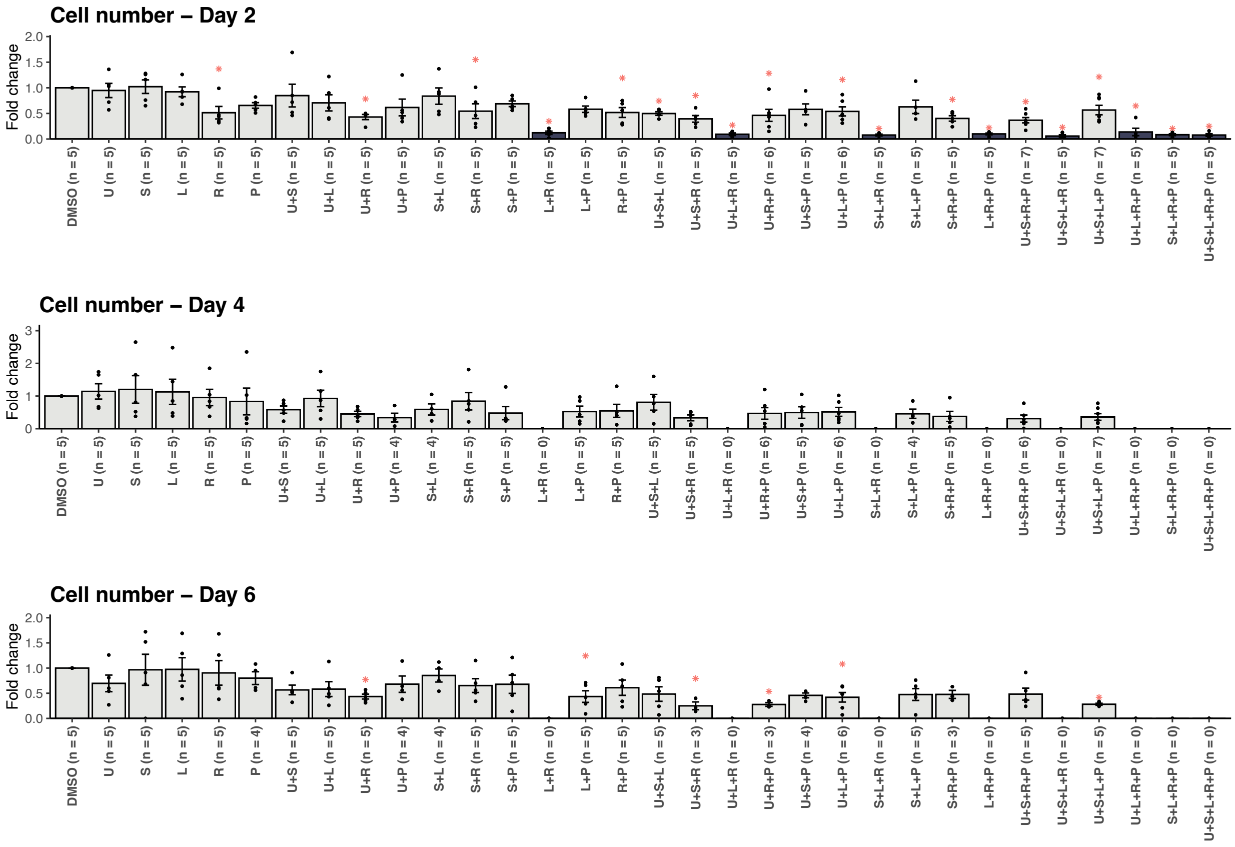

Fig. S3. Cell number data by inhibitor combination. Naïve ESCs were cultured over 6 days in 330 U/mL LIF + 400 µM L-proline with combinations of five inhibitors (U: U0126; S: SU5402; L: LY294002; R: rapamycin; P: PF-4708671). Cells were counted at passaged on day 2, 4, and 6. Conditions containing L+R (navy) were considered non-viable at day 2 and no data is available for this combination at days 4 and 6. Data is shown as mean and SEM with individual data points. Data were analysed using one-way ANOVA with Dunnett's multiple comparisons test to cells grown in 330 U/mL LIF+ 400 µM L-proline + DMSO, *$P < 0.05$.

Development • Supplementary information

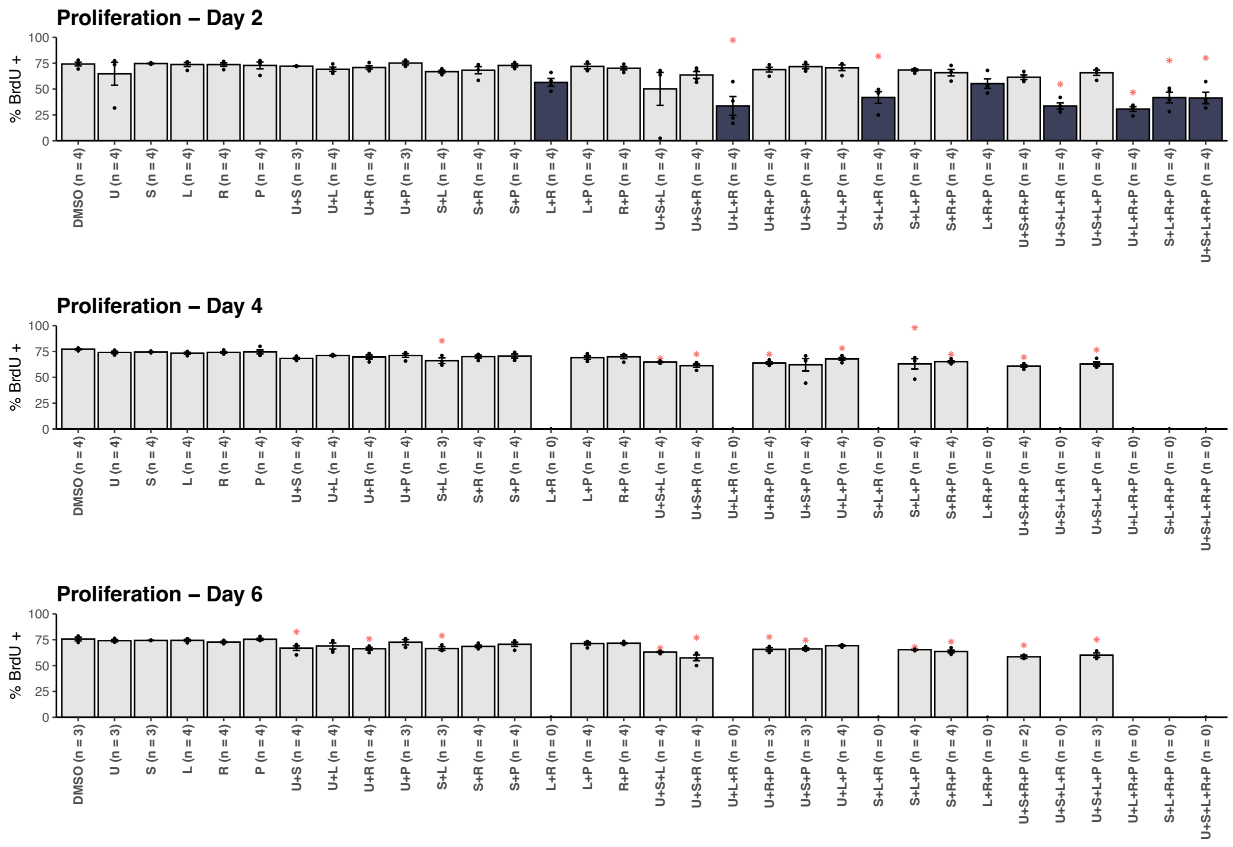

Fig. S4. Proliferation data by inhibitor combination. Naïve ESCs were cultured over 6 days in 330 U/mL LIF + 400 μM L-proline with combinations of five inhibitors (U: U0126; S: SU5402; L: LY294002; R: rapamycin; P: PF-4708671). Cells were passaged at day 2, 4, and 6, and proliferation was measured by BrdU incorporation and the percentage of cells in s-phase was quantified with flow cytometry. Conditions containing L+R (navy) were considered non-viable at day 2 and no data is available for this combination at days 4 and 6. Data is shown as mean and SEM with individual data points. Data were analysed using one-way ANOVA with Dunnett's multiple comparisons test to cells grown in 330 U/mL LIF+ 400 μM L-proline + DMSO, *$P < 0.05$.

Development • Supplementary information

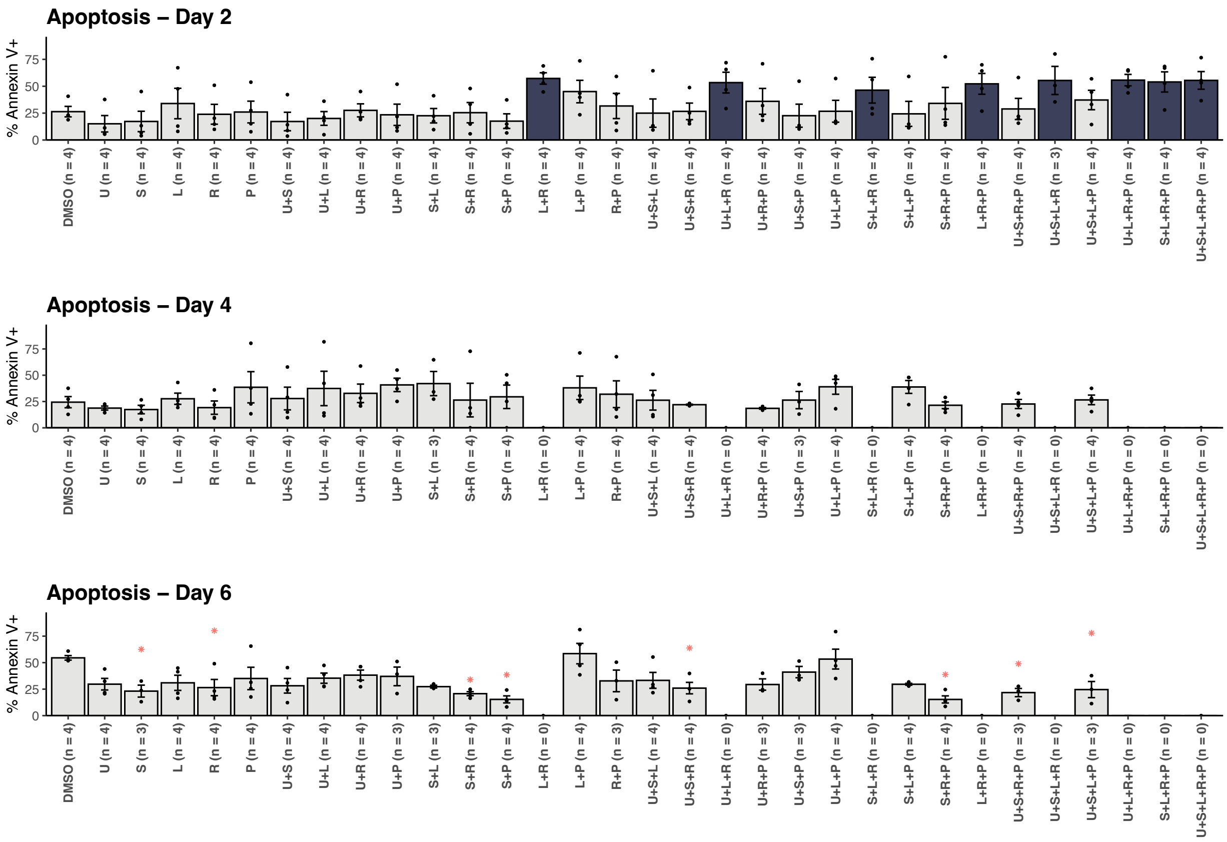

Fig. S5. Apoptosis data by inhibitor combination. Naïve ESCs were cultured over 6 days in 330 U/mL LIF + 400 μM L-proline with combinations of five inhibitors (U: U0126; S: SU5402; L: LY294002; R: rapamycin; P: PF-4708671). Cells were passaged at day 2, 4, and 6, and cells apoptosis and proliferation weas measured by Annexin V assay and quantified with flow cytometry. Conditions containing L+R (navy) were considered non-viable at day 2 and no data is available for this combination at days 4 and 6. Data is shown as mean and SEM with individual data points. Data were analysed using one-way ANOVA with Dunnett's multiple comparisons test to cells grown in 330 U/mL LIF+ 400 μM L-proline + DMSO, *$P < 0.05$.

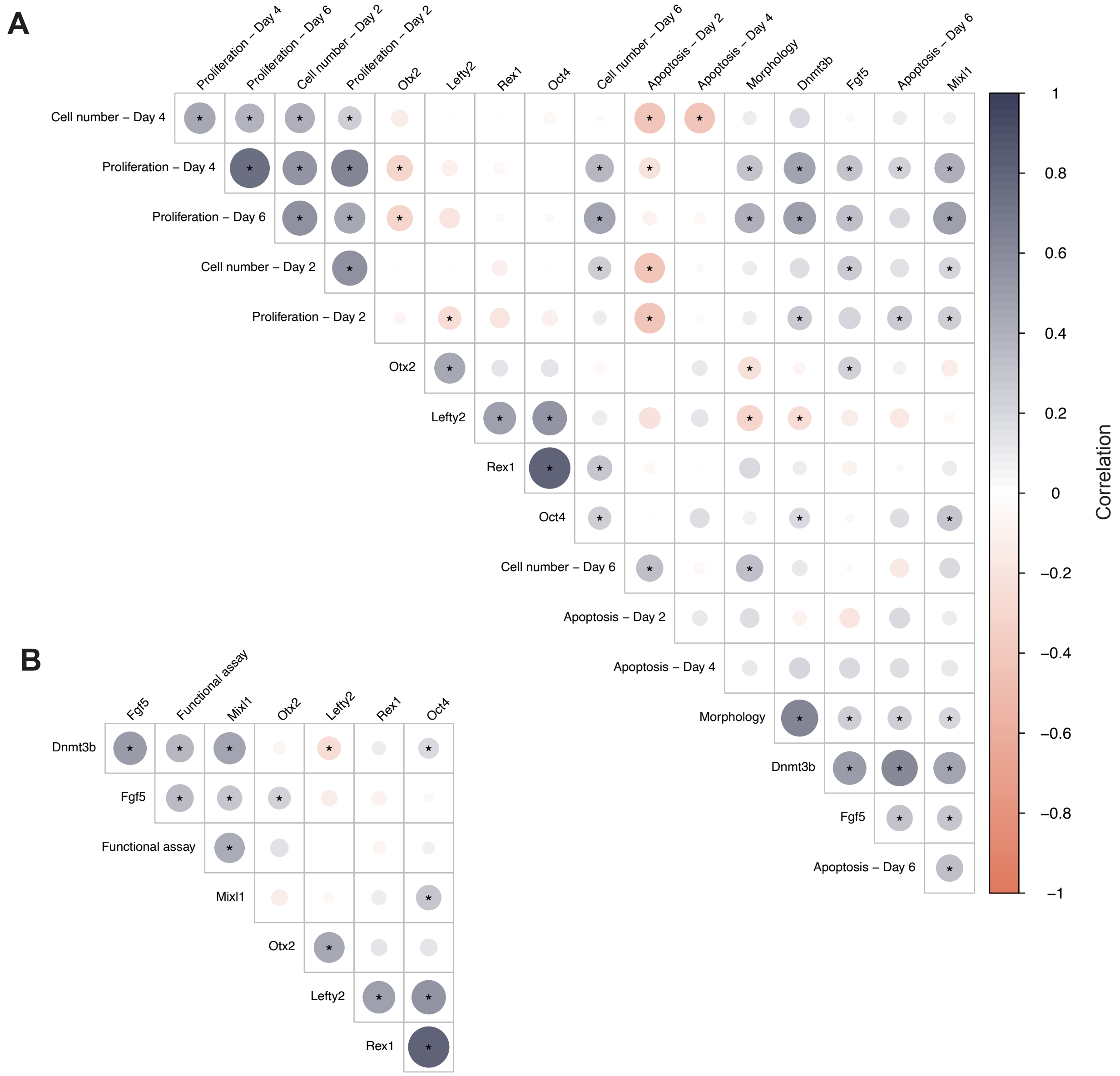

Fig. S6. Correlation matrix illustrates relationships between properties. ESCs were cultured to EPL cells in the presence of each inhibitor combination and assayed for emergent properties (cell number, proliferation, apoptosis, morphology) and gene expression, at the days shown. **A.** Correlation matrix of all parameters (n ≥ 3), sorted by hierarchical clustering. **B.** The correlation matrix was generated using a subset of the data in panel A, along with paired data from the functional assay. Functional assay data was included as cumulative *Brachyury* expression. Dot size and colour indicate the strength of either a positive (navy) or negative (red) correlation.

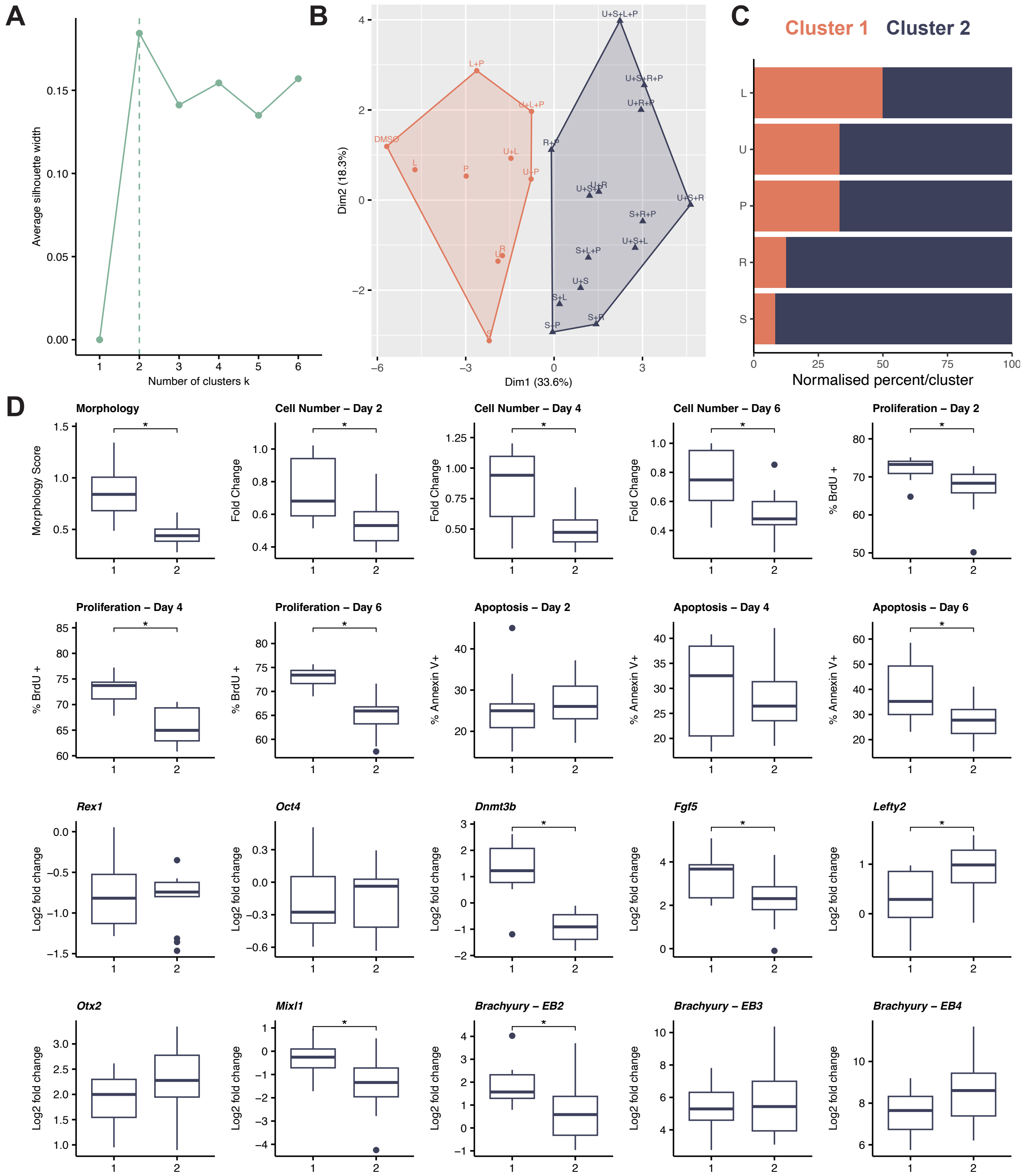

Fig. S7. Kmeans clustering reveals ESC-like and EPL-like clusters. ESCs were cultured to EPL cells in the presence of each inhibitor combination and assayed for emergent properties (cell number, proliferation, apoptosis, morphology) and gene expression, at the days shown. Data from each parameter were Z-scored and then subject to kmeans clusters. **A.** Silhouette score was calculated to inform the number of clusters in the dataset**. B.** Two kmeans clusters were visualised via principal component analysis (PCA). **C.** The frequency of the presence of each inhibitor was calculated and normalised to the number of conditions containing the inhibitor. **D.** Box plots showing the differences in each cluster. Statistical significance was calculated using a two-sided T-test with significance is denoted as *P < 0.05.

Development • Supplementary information

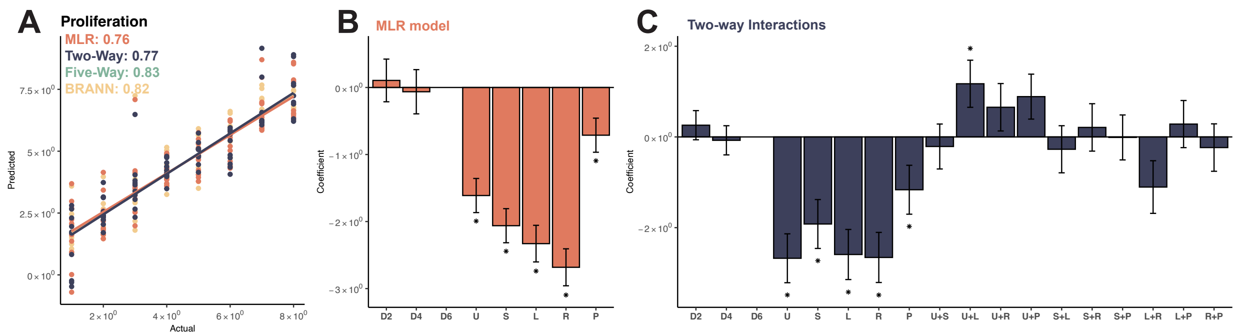

Fig. S8. Alternative model for proliferation data. Naïve ESCs were cultured over 6 days in 330 U/mL LIF + 400 μM L-proline with combinations of five inhibitors (U: U0126; S: SU5402; L: LY294002; R: rapamycin; P: PF-4708671). At days 2, 4 and 6 proliferation was measured. Data was averaged across biological replicates where n ≥ 3. To correct bimodal input data, data was binned into octiles each representing an equal proportion of the data. **A.** Data was modelled using either MLR, MLR with two-way interaction terms or a BRANNGP. Fit of each model is shown comparing the actual fit with the prediction from the model. **B.** Coefficients for each variable ± SEM for standard MLR. **C.** Coefficients for each variable ± SEM for MLR with two-way interaction terms. Significance is denoted as *$P < 0.05$.

Development • Supplementary information

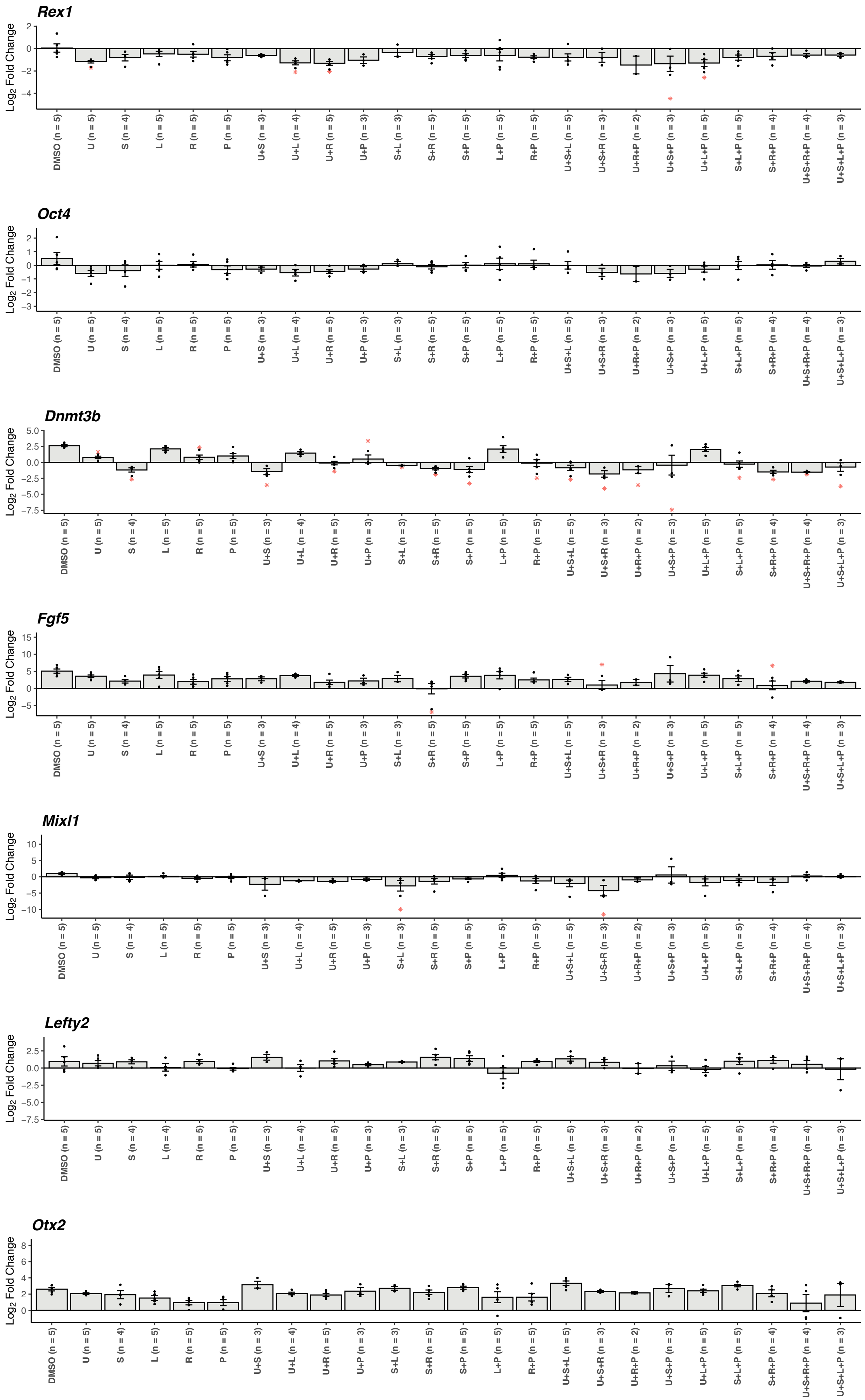
Rex1
Oct4
Dnmt3b
Fgf5
Mixl1
Lefty2
Otx2
Log2 Fold Change
DMSO (n = 5)
U (n = 5)
S (n = 4)
L (n = 5)
R (n = 5)
P (n = 5)
U+S (n = 3)
U+L (n = 4)
U+R (n = 5)
U+P (n = 3)
S+L (n = 3)
S+R (n = 5)
S+P (n = 5)
L+P (n = 5)
R+P (n = 5)
U+S+L (n = 5)
U+S+R (n = 3)
U+R+P (n = 2)
U+S+P (n = 3)
U+L+P (n = 5)
S+L+P (n = 5)
S+R+P (n = 4)
U+S+R+P (n = 4)
U+S+L+P (n = 3)

Fig. S9. Gene expression by inhibitor combination. Naïve ESCs were cultured over 6 days in 330 U/mL LIF + 400 µM L-proline with combinations of five inhibitors (U: U0126; S: SU5402; L: LY294002; R: rapamycin; P: PF-4708671). At day 6, cells were collected and analysed with qRT-PCR for pluripotency genes (*Rex1* and *Oct4,* primitive ectoderm markers (*Dnmt3b, Fgf5, Lefty2* and *Otx2*), and mesendoderm *genes (Mixl1*). Data is normalised to *β*-Actin and to cells grown in 1000 U/mL LIF. No data is available for conditions containing L+R as they were considered non-viable at day 2. Data is shown as mean log2 fold change and SEM with individual data points.

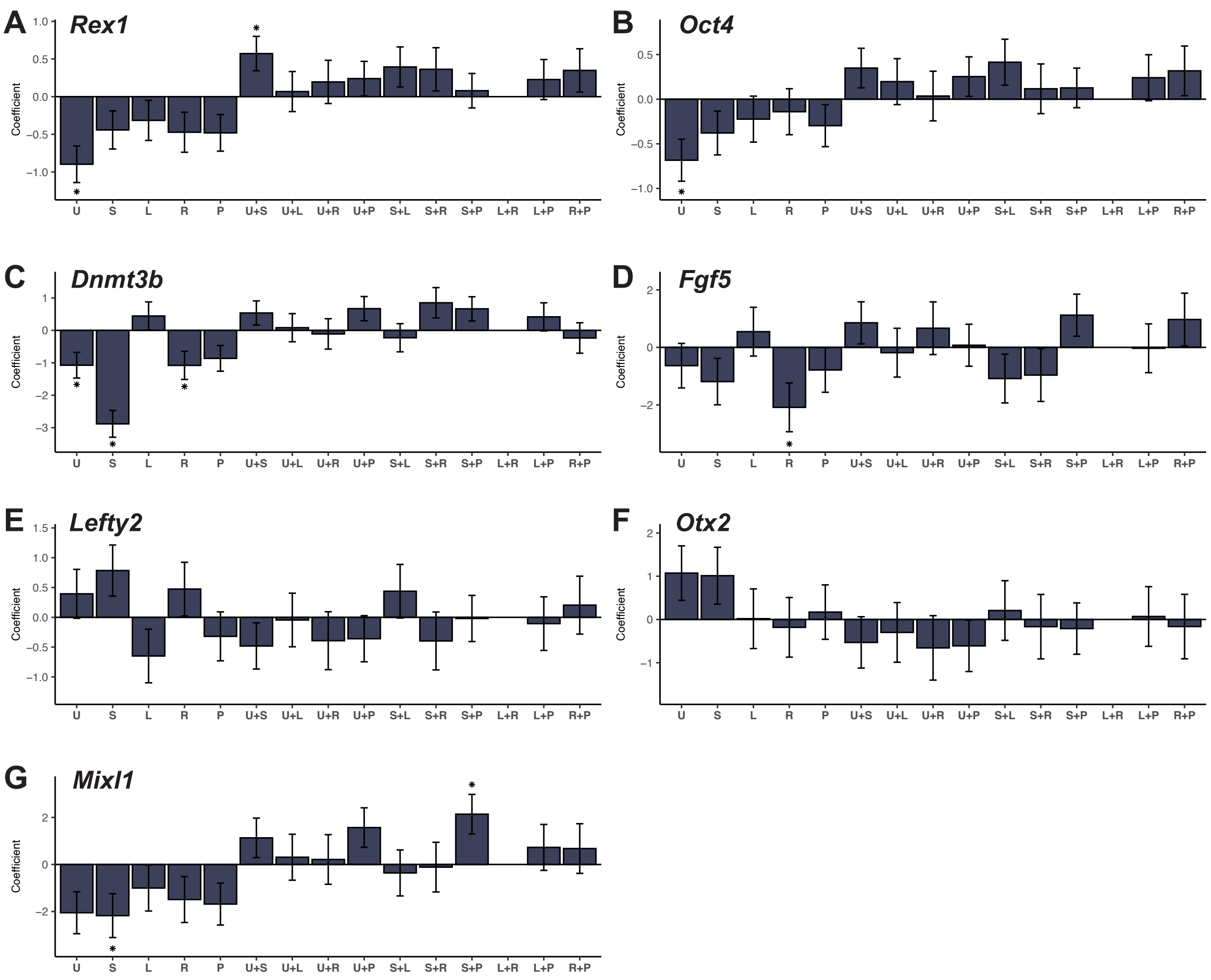

Fig. S10. Two-way interaction models for gene expression data. Naïve ESCs were cultured over 6 days in 330 U/mL LIF + 400 μM L-proline with combinations of five inhibitors (U: U0126; S: SU5402; L: LY294002; R: rapamycin; P: PF-4708671). At day 6, changes in expression of pluripotency genes (*Rex1*, **A** and *Oct4*, **B**), primitive ectoderm markers (*Dnmt3b*, **C***, Fgf5*, **D***, Lefty2*, **E** and *Otx2*, **F**), and mesendoderm *genes (Mixl1*, **G**). All samples were normalised to *β*-Actin and then to cells grown in 1000 U/mL LIF. Data shown is for n ≥ 3 biological replicates. Data was modelled using MLR with two-way interaction terms. Data shows coefficients for each inhibitor ± SEM. Significance is denoted as *P < 0.05.

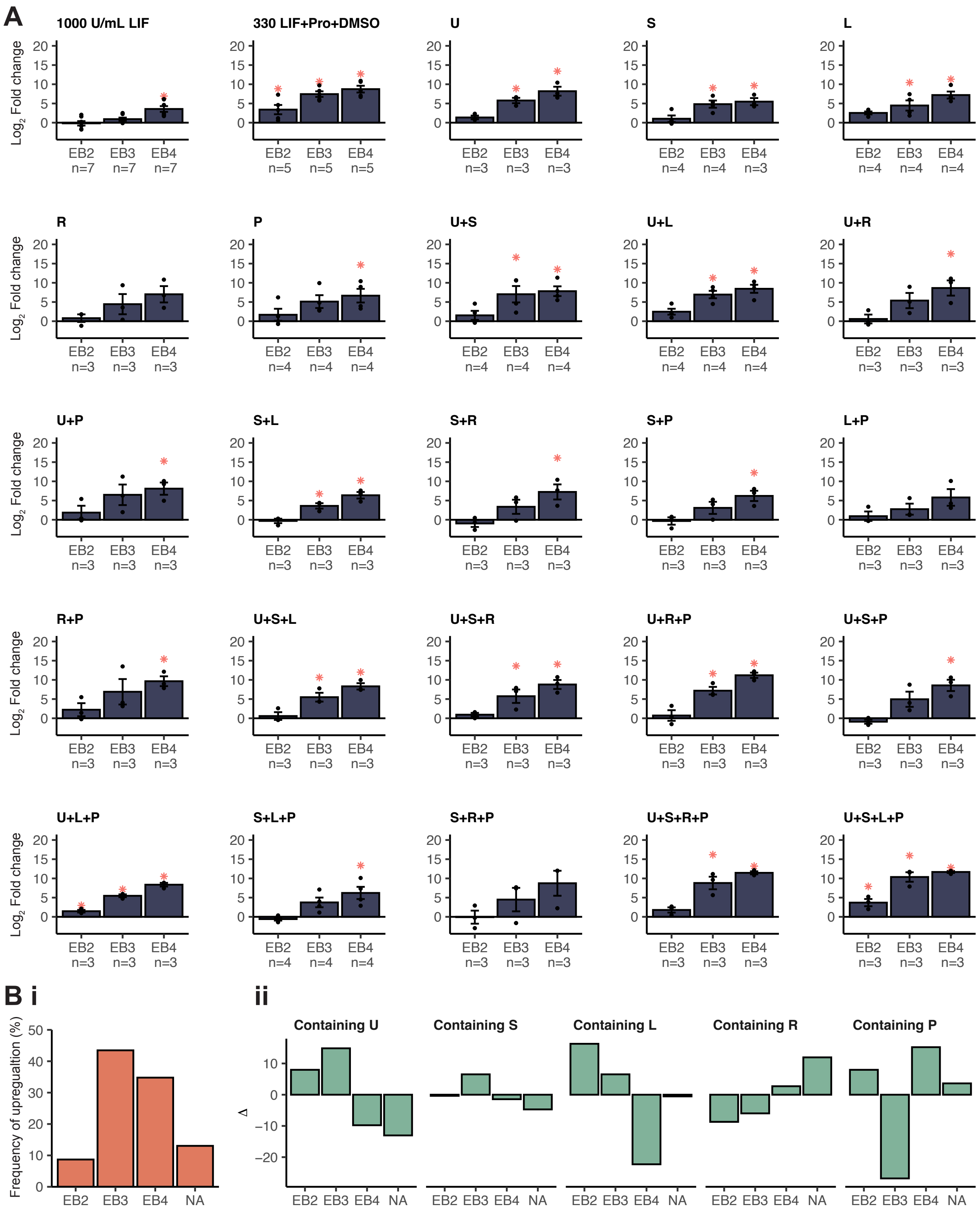

Development • Supplementary information

Fig. S11. Functional assay to determine position on the pluripotency continuum. Naïve ESCs were left maintained in 1000 U/mL LIF or cultured over 6 days in 330 U/mL LIF + 400 µM L-proline with combinations of five inhibitors (U: U0126; S: SU5402; L: LY294002; R: rapamycin; P: PF-4708671). Day 6 cells were spontaneously differentiated as embryoid bodies (EBs) on low adhesion plates in 0 U/mL LIF. mRNA samples were taken on day 2, 3 and 4 (EB2-4). Samples were analysed using qRT-PCR for *Brachyury* expression, a marker for the primitive streak. All samples were normalised to *β-Actin* as the reference gene and then to naïve ESCs. **A.** Mean log2 fold changes are shown ± SEM with individual data points. Data were analysed using a one-way ANOVA with *posthoc*Dunnett's multiple comparison test to naïve ESCs. Significance is denoted as *$P <$ 0.05. **Bi.** Frequency of the first significant upregulation across all inhibitor treated conditions. **Bii.** The change in frequency of first significant upregulation sorted for each condition.

Development • Supplementary information

Table S1. Summary of model fits. #Shapiro-Wilks test for normality of input data, and of residuals. If input data is < 0.05 then a transformation is preferential. If residuals are < 0.05, a linear fit may not be appropriate. Data in red does not pass the Shapiro-Wilks test, and alternate models should be considered. * Indicates that parameters are not relevant for the model style, but have been calculated for completeness. Sigma (**σ**) indicates standard error.

Available for download at
https://journals.biologists.com/dev/article-lookup/doi/10.1242/dev.201704#supplementary-data

Table S2. Comparison of linear models using F-test. P-values of comparison between models. A P < 0.05 indicates that the more complex model better explains the data.

Available for download at
https://journals.biologists.com/dev/article-lookup/doi/10.1242/dev.201704#supplementary-data

Table S3. Primers used for qRT-PCR

Available for download at
https://journals.biologists.com/dev/article-lookup/doi/10.1242/dev.201704#supplementary-data

Table S4. Solutions and buffers used to run and stain western blots.

Available for download at
https://journals.biologists.com/dev/article-lookup/doi/10.1242/dev.201704#supplementary-data

Table S5. Antibodies used in western blot staining. All antibodies were diluted in LI-COR Odyssey Blocking Buffer with 0.1% (v/v) Tween 20. All antibodies were supplied by Cell Signaling Technologies unless otherwise stated. DSHB: Developmental Studies Hybridoma Bank, SCB: Santa Cruz Biotechnology

Available for download at
https://journals.biologists.com/dev/article-lookup/doi/10.1242/dev.201704#supplementary-data

Table S6. Splitting data for training and test data. For each input dataset, we trialled splitting data into 80% training data to train the MLR and 20% testing data to check the predictive capacity of the model. Each dataset was subject to 50 random splits, and the mean, median, minimum and maximum of each the training and test adjusted R^2 and standard error (σ), and the distribution of significant variables from the training model.

Available for download at
https://journals.biologists.com/dev/article-lookup/doi/10.1242/dev.201704#supplementary-data
