# Supplementary material for: Signalling pathway crosstalk stimulated by L-proline drives mouse embryonic stem cells to primitive-ectoderm-like cells
Source: Development. 2023 Oct 26;150(20):dev201704. doi: 10.1242/dev.201704 (PMC10652046; doi:10.1242/dev.201704)
Supplement: Supplementary file 1 [file develop-150-201704_review_history.pdf]

Signalling pathway crosstalk stimulated by L-proline drives mouse embryonic stem cells to primitive-ectoderm-like cells

Hannah J Glover, Holly Holliday, Rachel A Shparberg, David Winkler, Margot Day and Michael B Morris

DOI: 10.1242/dev.201704

Editor: Jared Rutter

Review timeline

Original submission: 14 February 2023
Editorial decision: 2 May 2023
First revision received: 5 August 2023
Editorial decision: 11 September 2023
Second revision received: 27 September 2023
Accepted: 2 October 2023

Original submission

First decision letter

MS ID#: DEVELOP/2023/201704

MS TITLE: Signalling pathway crosstalk stimulated by L-proline drives differentiation of mouse embryonic stem cells to primitive-ectoderm-like cells

AUTHORS: Hannah J Glover, Holly Holliday, Rachel A Shparberg, David Winkler, Margot Day, and Michael B Morris

I have now received all the referees' reports on the above manuscript, and have reached a decision. The referees' comments are appended below, or you can access them online: please go to BenchPress and click on the 'Manuscripts with Decisions' queue in the Author Area.

As you will see, the referees express considerable interest in your work, but have some significant criticisms and recommend a substantial revision of your manuscript before we can consider publication. If you are able to revise the manuscript along the lines suggested, which may involve further experiments, I will be happy receive a revised version of the manuscript. Your revised paper will be re-reviewed by one or more of the original referees, and acceptance of your manuscript will depend on your addressing satisfactorily the reviewers' major concerns. Please also note that Development will normally permit only one round of major revision. If it would be helpful, you are welcome to contact us to discuss your revision in greater detail. Please send us a point-by-point response indicating your plans for addressing the referee's comments, and we will look over this and provide further guidance.

Please attend to all of the reviewers' comments and ensure that you clearly highlight all changes made in the revised manuscript. Please avoid using 'Tracked changes' in Word files as these are lost in PDF conversion. I should be grateful if you would also provide a point-by-point response detailing how you have dealt with the points raised by the reviewers in the 'Response to Reviewers' box. If you do not agree with any of their criticisms or suggestions please explain clearly why this is so.

Reviewer 1

© 2023. Published by The Company of Biologists under the terms of the Creative Commons Attribution License (https://creativecommons.org/licenses/by/4.0/).

Advance summary and potential significance to field

Glover and colleagues analysed the signalling pathways underpinning the pluripotency continuum from naïve ESC to EPL to peri-gastrulation states (by forming embryoid bodies) and more generally surveying the role of L-Proline in this conversion.

The authors characterised the signalling pathways involved in the maintenance of EPL cells. They have done so by performing comprehensive study on phosphorylation of 5 pathways across time, and by perturbing the pathways using inhibitors.

There is an important body of work including many western blots, qRT-PCR and image analysis, which are appropriate read-outs for this study. Computational analyses unveil relationships among the signalling pathways. Novel insights could be gleaned regarding the dynamic and synergy of these pathways during pluripotency dismantling and of the role of L-Proline in pluripotency maintenance, which could be of interest to stem cell biologists.

Comments for the author

I would recommend major revisions before publications. The data are here but the figures and text need to be clarified and simplified.

General comments:

It was very difficult to navigate the results. I added a lot more details below but overall all figures (except maybe Figure1) need to be clarified. Since a lot of conditions are tested, finding the key results is extremely tedious. I believe it is the responsibility of the authors to clarify their message. Also it appears that protocols might not always be consistent between analysis. It is not a problem but increases the complexity of the data.
The computational models developed later to assess inhibitors interactions is lacking justification. It would be appreciated if the authors could explain why they used these models rather than dimension reduction like PCA or tSNE which could also indicate parameters that are correlated.
In addition, to improve the clarity of the manuscript, the BRANNGP model should be removed. It's not used for analysing the relationships among pathways, so it is not useful to keep it – to me it's only adding confusion.
Finally, the authors need to share their raw data. Western blots (at least the intensity quantification), images scoring and table of Ct value are needed for anyone to reprocess the data (which was impossible for this round of review).
The code was not accessible (even on the bioRXiv version: https://www.biorxiv.org/content/10.1101/2023.02.14.528585v1).
In conclusion, the work presented here is promising. There are interesting results, but their presentation and then interpretation is too confusing. I would suggest major revisions of the figures and text according to the details below.

Detailed major issues:

Related to Figure 2:
• Figure 2B and C: It is quite surprising that p-Fgfr is the only significant change in the test performed. Additionally, the p-Fgfr blot is the one with the lowest contrast suggesting a reduced quality of the staining that may have led to a false positive.
• Figure 2B and C: Fgf and Erk have a similar trend on the blot (Figure 2B row 2 and 3, with same b-tubulin), which is expected. Quantification seem to show an increase of both signalling with time. But the colour scheme on Figure 2C is misleading because pErk is normalised to 0h staining which is high compare to pFgfr, therefore lead to a green colour compare to pFgfr which is blue, thus appear positive. It would be more interesting to show curves than squares (true for Figure 2E and F as well). Data are linked in time, so a plot going this way would be more interesting, and help reading the western blot rather than creating confusion. For instance, pStat3-Y705 is constantly increasing on quantification which is not visible on the blot. A curve would illustrate this very easily.

© 2023. Published by The Company of Biologists under the terms of the Creative Commons Attribution License (https://creativecommons.org/licenses/by/4.0/).

• There are not enough details in the methodology section. How are the statistics performed? On the figure legend it is indicated that Dunett's test was performed, but how many replicates of each Western blot were used for this analysis is not indicated. To this regard, why using Tukey for Figure 2D and Dunnett for C, E and F?
• I would include a supplementary figure showing the western blot and how the bands were selected for analysis. Some blots have several bands, which one has been selected for quantification? Maybe just include the quantification in the main results (similar to figure 2E and F), as having them side by side is very difficult to interpret as illustrated above. Put the western blot images in Supplementary figures. There are no blots images for Figure 2E and 2F. Quantification data needs to be added as a supplementary table for anyone to be able to redo the statistical analysis.
• From figure legend, one could guess:
o Figure 2B: 330U/mL LIF + L-Proline (no concentration indicated, likely 400uM) -> line 615
o Figure 2D: DMEM + 0.1%FBS + 1mM L-proline (LIF?) -> Line 618
o Figure 2E: 1000U/mL LIF + 400uM L-Proline -> Line 620
o Figure 2F: 400uM L-Proline (LIF?) -> Line 622
Why using different protocols especially serum starvation? Could you indicate the different protocols in the figure, maybe on top of the western blots' quantification? Also mention it in the results section, it is only present in the figure legend.
• Line 136: "L-proline acutely increased phosphorylation of Erk1/2 and Stat3Y705 in these conditions (Fig. 2D)." -> you must mention in the text that the culture conditions are not the same.
• Line 158: "pRps6 phosphorylation was suppressed for up to 10 h by rapamycin and LY294002 (0.26 ± 0.02 SEM, Fig. 2E)." First, Fig2E shows a downregulation of pRps6 under L treatment close to -2. Maybe its Fig2Fii but the suppression of pRsp6 with L treatment is no longer significant at 10h, so I wonder what this number is referring to. Both results are inconsistent anyway maybe due to different protocols.

Related to Figure 3, 4, S2, S3 and S5:
• Line 175: Maybe break the Figure S2 panel into sub panels to facilitate the reading. Highlighting the bar plot was a good idea. Maybe bold the labels as well to make it clearer.
• Figure S2: Why no statistics? The legend does not mention any test.
• Line 178 to 188: So, correlation and anticorrelation were found, but you must describe these results to greater length.
• Line 213: "BRANNGP models with improved fit suggest additional nonlinear factors are involved. However, the nature and magnitude of these factors cannot as yet be deconvoluted from the BRANNGP models." Here it's a bit confusing.
BRANNGP fits the data better but it is not used later in the analysis, is the statement above the reason why BRANNGP was not retained? The manuscript would benefit in clarity if this model was not mentioned. Linear and two-way MLR are sufficient to explain synergy etc…
• Line 689: "The sum of the three days of Brachyury data was used as a proxy for the slope." I do not understand this sentence. Maybe explain in more details in the method section
• Figure S5: Why no statistics? The legend does not mention any test.
Before performing regression model, it would be worth indicating which genes are statistically up or down regulated.

Related to Figure S7:
• Figure S7Bi: What is the goal of this piece of data? Why summarising all conditions?

Discussion:
• Line 385: "Collectively, these results highlight biological system complexity and make it difficult, if not impossible, a priori to determine outcomes even when a single inhibitor is used." Rather than saying it's impossible I would suggest some follow up experiments required to answer this question. Transcriptomic analysis might be an option.
• Line 414: "No significant changes in expression of Mixl1 were observed either in the absence or presence of the inhibitors (Fig. 1Fii, 4G), indicating that cells did not form mesendoderm." According to the data this statement is wrong. The dichotomy between Mixl1 and Brachyury expression comes from the fact that the authors do not compare the same cell types. Cells did not undergo mesendoderm differentiation prior to EB as they are maintained in 300U/mL LIF + Pro (Fig1ii and Fig4G). Only in 0U/mL LIF, for 4 days show a increased Mixl1 expression. Brachyury is

© 2023. Published by The Company of Biologists under the terms of the Creative Commons Attribution License (https://creativecommons.org/licenses/by/4.0/).

expressed in EBs (and not tested in 300U/mL LIF + Pro) which are Day8 to Day12 (6 Days of 300U/mL LIF + Pro then 2 to 4 days as EBs).
It would be surprising that EBs do not express Mixl1 but this was not assessed.
In conclusion, Brachyury and Mixl1 expression data cannot be compared in this study.

Minor issues:
• Line 127: Pluripotence -> pluripotency
• Line 149: is it U0126 or UO126 like in Figure 2D
• Line 294: There is no Figure 7Bii -> Figure S7Bii
• Line 409 and 411: Figure S7
• Line 446: though -> thought
• Line 524: For data in Fig. 1D -> it seems to be wrong as Fig 1D is about apoptosis and not about phosphorylation of signalling pathway. Is it related to Fig2D?
• Line 563: The link to the R code did not work on the PDF, so I could not check how data were analysed.
• Figure 3B to E: Could you indicate the models on top of panels (ii) and (iii). This information is only available in Figure S4.

Reviewer 2

Advance summary and potential significance to field

In the manuscript entitled 'Signaling Pathway Crosstalk Stimulated by L-Proline Drives Differentiation…', Glover and coworkers confirmed and extended previous observations that the availability of proline modulates different signaling pathways in ESCs. They used an innovative approach to understand the pathways that control proline-induced effects (including morphological transition, cell proliferation/apoptosis, and expression of a few marker genes), and determined whether these pathways act antagonistically or synergistically. The results are very interesting and provide insights into the complexity of the early phase of the developmental process. However, in my opinion, prior to publication, it is necessary to make some clarifications/revisions regarding the terminology used to describe the effects of proline supplementation on ESC behavior.

Comments for the author

Major points:

1) differentiation vs naïve to early primed transition In some passages of this manuscript the authors claim that proline supplementation induces ESC differentiation:
Title
….drives differentiation of mouse embryonic stem cells to primitive-ectoderm-like cells
Abstract
sentence 'and each inhibitor blocked specific aspects of differentiation'.
Introduction
pag 2. line 38: sentence 'stimulates differentiation to a second pluripotent population known as early primitive ectoderm-like cells (EPL cells)'
pag. 2 line 55: sentence 'The continued presence of L-proline in culture then drives EPL cells to neural cells by a series of embryologically relevant intermediate cell types'.
Rathejn and co-workers demonstrated that EPL cells are more prone to differentiate (doi: 10.1242/dev.129.11.2649). Proline, on the other hand, does not induce neural differentiation. It is worth noting that recent reports indicate that 3D aggregates of EPL/PiCs elongate earlier than aggregates of naïve cells in gastruloid formation assays (Cermola et al. 2021, doi: 10.1016/j.stemcr.2020.12.013).
pag.3 line 58:. sentence. 'L-proline stimulates development/differentiation include..'
pag.3 line 62:..sentence…. 'that drive developmental progression and differentiation..'
Results
pag. 4 line 93: sentence ..'and then directed to differentiate into EPL cells by addition of 400 μM L-proline for 6 days'.
pag. 4 line 101: sentence ..' at days 2, 4 and 6 of differentiation…'

© 2023. Published by The Company of Biologists under the terms of the Creative Commons Attribution License (https://creativecommons.org/licenses/by/4.0/).

pag. 4 line 111: sentence ..‘ After 6 days of differentiation…’ it would be better to use ‘After 6 days of incubation’
pag. 6 line 167: sentence. …‘was designed for the differentiation of ESCs to EPL’
pag. 9 line 286: sentence. …‘ After 6 days of differentiation…’
Discussion
pag. 10 line 301: sentence ..‘ during ESC differentiation to EPL cells’
pag. 10 line 303: sentence ..‘ that mediate self-renewal, differentiation,’
pag. 10 line 309: sentence ..‘ associated with proline-mediated differentiation to EPL cells,’
pag. 10 line 315: sentence ..‘ the course of differentiation, Fgfr phosphorylation increased,’
pag. 11 line 337: sentence ..‘ during ESC differentiation to EPL cells,’
pag. 12 line 366: sentence ..‘ L-proline in promoting directed differentiation,’
pag. 13 line 392: sentence ..‘ in differentiation to EPL cells,’
pag. 14 line 447: sentence ..‘ mechanisms of L-proline-mediated differentiation…,’
pag. 14 line 456: sentence ..‘ differentiation of pluripotent cells towards neuroectoderm…,’
pag. 15 line 458: sentence ..‘ proline mediated differentiation provides a useful model…,’
Methods
pag.15 line 470: sentence ‘ESCs were differentiated to EPL
pag.15 line 481: sentence ‘At days 2, 4 and 6, differentiating ESCs treated with 1000 u/mL LIF or 330 U/mL LIF,’
When cultured with LIF, the cells are unable to differentiate. It is worth noting that EPL cells are dependent on LIF for their survival and proliferation.
Figure legends
pag. 21 line 660: sentence ‘control different aspects of differentiation..’

In other passages the authors claim that proline supplementation induces a pluripotency state transition:
Abstract
sentence ‘drives mouse embryonic stem cells (ESCs) to a transcriptionally distinct pluripotent cell population - early primitive ectoderm-like (EPL) cells - which lies between the naïve and primed states’
Introduction
pag. 2 line 42. sentence ‘EPL cells/PiCs are metastable, as they revert to naïve ESCs upon removal of L-proline’.
It is known that differentiated cells are unable to revert spontaneously to the naïve state of pluripotency pag. 2 line 48. sentence ‘EPL cells are more primed to differentiate than ESCs’.
Results
pag. 4 line 115. sentence ….‘indicating maintenance of pluripotency’.

Previous studies have demonstrated that EPL/PiCs cells generated in DMEM/FBS/LIF medium supplemented with proline are pluripotent stem cells. Evidence from both phenotypic (blastocyst injection/chimera, teratoma, and gastruloid formation), and molecular (transcriptome and global methylation profiling) assays, support the idea that a high proline regimen drives naïve ESCs towards an intermediate/early primed transitional state between naïve and primed pluripotency states. Moreover, EPL/PiCs cells have been shown to be pluripotent undifferentiated cells (doi: 10.3390/CELLS11142125) and competent at primordial germ cell differentiation (Cermola et al. 2021, doi: 10.1016/j.stemcr.2020.12.013). Notably, EPL cells are capable of spontaneously reverting to the naïve state, while differentiated cells are unable to do so.
In my opinion, proline cannot be considered an inducer of ESC differentiation. Moreover, the resulting EPL/PiCs cells, which are captured in a stable intermediate state of pluripotency, cannot be defined as differentiated cells.

2) the role played by Snat2
In the schemes shown in Fig. 2 and Fig. 5, the modulation of Stat, mTor, Pi3K, and Mek signaling pathways by a high proline regimen is dependent on proline uptake through Snat2. However, no experimental evidence regarding the role of this specific amino acid channel in proline-mediated modulation of the signaling pathways is presented here. Nevertheless, it is highly plausible that this is the case.

Minor points:

© 2023. Published by The Company of Biologists under the terms of the Creative Commons Attribution License (https://creativecommons.org/licenses/by/4.0/).

The AAR pathway was investigated through transcriptome analysis, in combination with pharmacological (specific inhibitor of the prolyl-tRNA synthetase, Halofuginone) and genetic approaches (downregulation and ectopic expression of ATF4). The results obtained supported the idea that proline supplementation induces alleviation of the amino acid stress response (AAR) pathway, specifically the GCN2-ATF4 signaling pathway (D'Aniello et al., 2015, DOI: 10.1038/cdd.2015.24).

The TGF-β pathway has been shown to play a role in proline-induced primed states via autocrine FGF and TGF-β signaling pathways. Supplemental proline was found to induce Smad2 phosphorylation, as reported by D'Aniello et al. in 2017 (doi: 10.1016/j.stemcr.2016.11.011). Additionally, the presence of the TGF-β chemical inhibitor SB431542 impaired proline-induced phenotypic transition (D'Aniello et al., 2017, doi: 10.1016/j.stemcr.2016.11.011).

First revision

Author response to reviewers' comments

Dear Editors and Reviewers,

Thank you for reviewing this manuscript. We appreciate your valuable feedback. We have revised the manuscript to include these changes. Attached is a point-by-point response to each of the reviewers' comments. We have included line numbers where relevant, and we have also colour coded the responses from the reviewers in the main text of the paper (reviewer 1 comments in green and reviewer 2 comments in blue). Minor edits were made elsewhere to improve syntax but the data, conclusions and discussion points have not be changed.

We hope the revised manuscript provides the necessary clarity to communicate our research. We believe this is an important work to demonstrate how amino acids can activate a complex signalling network to alter gene expression and emergent properties. This mechanism contributes to understanding the transition between pluripotent states, and as a model for peri-implantation embryogenesis.

Thank you for your time and comments.

Sincerely,
Dr. Hannah Glover and Dr. Michael Morris, on behalf of all authors

Reviewer 1 Advance Summary and Potential Significance to Field:
Glover and colleagues analysed the signalling pathways underpinning the pluripotency continuum from naïve ESC to EPL to peri-gastrulation states (by forming embryoid bodies) and more generally surveying the role of L-Proline in this conversion.

The authors characterised the signalling pathways involved in the maintenance of EPL cells. They have done so by performing comprehensive study on phosphorylation of 5 pathways across time, and by perturbing the pathways using inhibitors.

There is an important body of work including many western blots, qRT-PCR and image analysis, which are appropriate read-outs for this study. Computational analyses unveil relationships among the signalling pathways. Novel insights could be gleaned regarding the dynamic and synergy of these pathways during pluripotency dismantling and of the role of L-Proline in pluripotency maintenance, which could be of interest to stem cell biologists.

Reviewer 1 Comments for the Author: I would recommend major revisions before publications. The data are here but the figures and text need to be clarified and simplified.

© 2023. Published by The Company of Biologists under the terms of the Creative Commons Attribution License (https://creativecommons.org/licenses/by/4.0/).

General comments:

It was very difficult to navigate the results. I added a lot more details below, but overall all figures (except maybe Figure1) need to be clarified. Since a lot of conditions are tested, finding the key results is extremely tedious. I believe it is the responsibility of the authors to clarify their message. Also, it appears that protocols might not always be consistent between analysis. It is not a problem but increases the complexity of the data. The computational models developed later to assess inhibitors interactions is lacking justification. It would be appreciated if the authors could explain why they used these models rather than dimension reduction like PCA or tSNE which could also indicate parameters that are correlated. In addition, to improve the clarity of the manuscript, the BRANNGP model should be removed. It's not used for analysing the relationships among pathways, so it is not useful to keep it – to me it's only adding confusion. Finally, the authors need to share their raw data. Western blots (at least the intensity quantification), images scoring, and table of Ct value are needed for anyone to reprocess the data (which was impossible for this round of review). The code was not accessible (even on the bioRxiv version: https://www.biorxiv.org/content/10.1101/2023.02.14.528585v1). In conclusion, the work presented here is promising. There are interesting results, but their presentation and then interpretation is too confusing. I would suggest major revisions of the figures and text according to the details below.

Author response:

Thank you for reviewing our manuscript. We appreciate your feedback. We have clarified the figures as per the reviewer suggestions below (i.e., bolding text, changing heatmaps to bar plots).

We have included a PCA analysis of our data matrix as Figure S7. Here we use kmeans clustering to distinguish two populations, and we can infer which of these inhibitors are best at producing cells most ES like, or conversely which inhibitors permit differentiation to EPL cells. We believe this nicely complements the regression modelling used in the main text of the paper, but this does not address how some inhibitors change some properties or genes and not others. Further, our two-way interaction effects could be used to glean even more information about how inhibitors work either synergistically or antagonistically.

We thank the reviewer for identifying a lack of clarity in what the nonlinear neural network models (BRANNGP) are used for. We have significantly revised the description of this method and results to make it clearer that there is substantial nonlinearity in the relationships between the factors and cell responses that are not picked up by the linear models, with or without interaction terms. The neural network automatically accounts for interactions an also nonlinearity. Its primary purpose is to more accurately predict the behaviour of stem cells for a given combination of factors. However, as the relevance of these factors in nonlinear models is local (context-dependent), it is not possible to provide a simple explanation like the linear model can. Thus, in summary, linear models are poorer predictors but better at explaining the effects of factors while nonlinear models are better predictors but are harder to interpret. This section has been rewritten to address these concerns and provide additional clarity (lines 200-235).

We apologise for the issue with the GitHub link – the repository was not set to be publicly available. After consulting with the editors, we have moved all of this data over to Zenodo as this provides non-editable code with a doi. This can be found at https://zenodo.org/record/8035085 and is included in the new Data availability section on lines 645-648.

After consultation with the editor, the processed data currently on Zenodo was deemed to meet the requirements. Raw data is available on request and has been indicated in the manuscript on line 646.

Detailed major issues:
Related to Figure 2:

- Figure 2B and C: It is quite surprising that p-Fgfr is the only significant change in the test performed. Additionally, the p-Fgfr blot is the one with the lowest contrast suggesting a reduced quality of the staining that may have led to a false positive.

© 2023. Published by The Company of Biologists under the terms of the Creative Commons Attribution License (https://creativecommons.org/licenses/by/4.0/).

- We believe that these signals are tightly controlled by signalling pathway cross talk, hence the requirement of multiple inhibitors to fully attenuate the response. Phospho-Fgfr antibodies are not widely available, and the antibody used was tried at several concentrations to optimize staining. The significant result was obtained from n=3 samples. Representative blots were included to provide full transparency of the strength of signalling (Fig S1).

- Figure 2B and C: Fgf and Erk have a similar trend on the blot (Figure 2B, row 2 and 3, with same β- tubulin), which is expected. Quantification seem to show an increase of both signalling with time. But the colour scheme on Figure 2C is misleading because pErk is normalised to 0h staining which is high compare to pFgfr, therefore leads to a green colour compared to pFgfr which is blue, thus appears positive. It would be more interesting to show curves than squares (true for Figure 2E and F as well). Data are linked in time, so a plot going this way would be more interesting, and help reading the western blot rather than creating confusion. For instance, pStat3-Y705 is constantly increasing on quantification which is not visible on the blot. A curve would illustrate this very easily.
    - We have updated the visualization from heat map to bar plots including error bars. We decided against a curve as this data was not collected continuously, and bar plots allow us to show the error. We believe this accomplishes the same goal while providing additional information.
- There are not enough details in the methodology section. How are the statistics performed? On the figure legend it is indicated that Dunett's test was performed, but how many replicates of each Western blot were used for this analysis is not indicated. To this regard, why using Tukey for Figure D and Dunnett for C, E and F?
    - We have updated the methods to give more details on the statistics (Lines 592-594, 604- 609). Full statistical code is also available on GitHub. Due to the nature of this figure, we are not able to show the n for each group, but we have included dots for each sample, and the number of replicates in this in the figure legend. Tukey was used for Figure D as this figure contained samples +/- L-proline as well as +/- U0126. Tukey provided us with the option to directly compare these groups, where this was not relevant for Figures C, E and F.
- I would include a supplementary figure showing the western blot and how the bands were selected for analysis. Some blots have several bands, which one has been selected for quantification? Maybe just include the quantification in the main results (similar to figure 2E and F), as having them side by side is very difficult to interpret as illustrated above. Put the western blot images in
    - We have modified Fig. 2 to show just the quantification, and we have modified Fig. S1 to include all western blots from figure 2, and have added representative western blots corresponding to the original Figure 2E & F. This figure also contains a panel showing how the quantitation was performed for blots with different numbers of bands. The methods section was also updated to include this information (lines 568-571).
- Supplementary figures. There are no blots images for Figure 2E and 2F. Quantification data needs to be added as a supplementary table for anyone to be able to redo the statistical analysis.
    - We have added representative blots for these experiments in Fig. S1. Normalized intensity values used to generate these plots in available on Zenodo.
- From figure legend, one could guess:
  Figure 2B: 330U/mL LIF + L-Proline (no concentration indicated, likely 400uM) -> line 615
  Figure 2D: DMEM + 0.1%FBS + 1mM L-proline (LIF?) -> Line 618
  Figure 2E: 1000U/mL LIF + 400uM L-Proline -> Line 620
  Figure 2F: 400uM L-Proline (LIF?) -> Line 622
  Why using different protocols especially serum starvation? Could you indicate the different protocols in the figure, maybe on top of the western blots' quantification? Also mention it in the results section, it is only present in the figure legend.
    - These culture conditions were used to assess different aspects of the signalling landscape. The first experiment (previously and currently 2B) was used to look at baseline changes in cell signalling across the transition from ES cells to EPL cells. The serum starvation protocol (previously 2D and currently 2C) uses a similar media composition but has a significantly lower FBS concentration for 4 hours. This reduces

© 2023. Published by The Company of Biologists under the terms of the Creative Commons Attribution License (https://creativecommons.org/licenses/by/4.0/).

basal cell activity and establishes a clear signal from when L-proline was added, and more effectively links L-proline to changes in cell signalling. The next panels (previously 2E&F and currently 2D&E) were intended to capture changes in L-proline-mediated cell signalling with one or more inhibitors. These conditions contained 1000 U/mL LIF, and 400 μM L-proline was added at 0 h. We have altered the figure, figure legend and results (lines 133-134, 144-146) to make this clearer.

- Line 136: "L-proline acutely increased phosphorylation of Erk1/2 and Stat3Y705 in these conditions (Fig. 2D)." -> you must mention in the text that the culture conditions are not the same.
  - Updated the text to include culture conditions (Lines 133-134, 144-146).
- Line 158: "pRps6 phosphorylation was suppressed for up to 10 h by rapamycin and LY294002 (0.26 ± 0.02 SEM, Fig. 2E)." First, Fig2E shows a downregulation of pRps6 under L treatment close to -2. Maybe its Fig2Fii but the suppression of pRsp6 with L treatment is no longer significant at 10h, so I wonder what this number is referring to. Both results are inconsistent anyway, maybe due to different protocols.
  - The visualization in the heatmap was Log2 normalized for ease of understanding, but the quantification in text refers to the fold-change. We have removed the confusing descriptive statistics, and timing discrepancy has been clarified in the text (line 157).

Related to Figure 3, 4, S2, S3 and S5:

- Line 175: Maybe break the Figure S2 panel into sub panels to facilitate the reading. Highlighting the bar plot was a good idea. Maybe bold the labels as well to make it clearer.
  - We have divided figure S2 into panels, and they are now listed as Fig S2 (Morphology), S3 (Cell number), S4 (Proliferation) and S5 (Apoptosis). Labels were changes to bold throughout the paper.
- Figure S2: Why no statistics? The legend does not mention any test.
  - These plots were intended to show the raw data which was used as inputs for the linear models and allow readers to look at specific combinations. Statistics were not initially included as we believe linear regression is the most powerful way to glean information while avoiding false positives which frequently occurs with so many combinations. We updated these figures and legends to include one-way ANOVA with Dunnett's multiple comparisons test to 330 U/mL LIF + 400 uM L-proline + DMSO.
- Line 178 to 188: So, correlation and anticorrelation were found, but you must describe these results to greater length.
  - We have updated the results to provide more detail including R^2 values (lines 179-187). We also included a PCA plot as previously suggested and used kmeans clustering to break the data into two clusters. These groups were further used to classify parameters with similar expression patterns. This is summarized in Fig. S7 and in the results section (lines 188-194).
- Line 213: "BRANNGP models with improved fit suggest additional nonlinear actors are involved. However, the nature and magnitude of these factors cannot as yet be deconvoluted from the BRANNGP models." Here it's a bit confusing. BRANNGP fits the data better but it is not used later in the analysis, is the statement above the reason why BRANNGP was not retained? The manuscript would benefit in clarity if this model was not mentioned. Linear and two-way MLR are sufficient to explain synergy etc…
  - See comprehensive explanation above and in the revised manuscript.
- Line 689: "The sum of the three days of Brachyury data was used as a proxy for the slope." I do not understand this sentence. Maybe explain in more details in the method section
  - This was changed to 'Functional assay data was included as cumulative *Brachyury* expression'. Essentially, we used the AUC for this data across the three timepoints to allow for differences in temporal expression of Brachyury. This is updated in the figure legend.
- Figure S5: Why no statistics? The legend does not mention any test. Before performing regression model, it would be worth indicating which genes are statistically up or down regulated.
  - These plots were intended to show the raw data which was used as inputs for the linear models, and allow readers to look at specific combinations. Statistics were not

© 2023. Published by The Company of Biologists under the terms of the Creative Commons Attribution License (https://creativecommons.org/licenses/by/4.0/).

initially included as we believe linear regression is the most powerful way to glean information while avoiding false positives which frequently occurs with so many combinations. We updated these figures to include one-way ANOVA with Dunnett's multiple comparisons test to 330 U/mL LIF + 400 uM L-proline + DMSO.

Related to Figure S7:

- Figure S7Bi: What is the goal of this piece of data? Why summarising all conditions?
    - o We wanted to show a histogram of when Brachyury was first upregulated, as this shows where these cells sit on the pluripotency continuum. This is a useful lead-in to S7Bii, which shows the distribution of the inhibitors based on this histogram. From this we can see that any combination with LY-294002 tended to upregulate Brachyury quickly (i.e., they were more primed), whereas combinations containing PF-4708671 tended to upregulate Brachyury later (i.e. they were more naïve). This explanation has been updated on lines 438-449.

Discussion:

- Line 385: "Collectively, these results highlight biological system complexity and make it difficult, if not impossible, a priori to determine outcomes even when a single inhibitor is used." Rather than saying it's impossible I would suggest some follow up experiments required to answer this question. Transcriptomic analysis might be an option.
    - o We have updated the discussion to provide more concrete examples (Lines 412-417) including mass spectrometry based phosphoproteomics and automated high throughput assays focused which could estimate whether cells have reached EPL cells using just one or two of the parameters which are highly correlated in this study.
- Line 414: "No significant changes in expression of Mixl1 were observed either in the absence or presence of the inhibitors (Fig. 1Fii, 4G), indicating that cells did not form mesendoderm." According to the data this statement is wrong. The dichotomy between Mixl1 and Brachyury expression comes from the fact that the authors do not compare the same cell types. Cells did not undergo mesendoderm differentiation prior to EB as they are maintained in 300U/mL LIF + Pro (Fig1ii and Fig4G). Only in 0U/mL LIF, for 4 days show a increased Mixl1 expression. Brachyury is expressed in EBs (and not tested in 300U/mL LIF + Pro) which are Day8 to Day12 (6 Days of 300U/mL LIF + Pro then 2 to 4 days as EBs). It would be surprising that EBs do not express Mixl1 but this was not assessed. In conclusion, Brachyury and Mixl1 expression data cannot be compared in this study.
    - o We believe that this sentence was poorly worded and placed ambiguously within a paragraph also containing information about the functional assay. This sentence was initially included to indicated that during the culture of ES to EPL cells in the presence of inhibitor combinations, there were no changes in Mixl1 expression. This has been rephrased and incorporated into the previous paragraph (lines 438-440).

Minor issues:

- Line 127: Pluripotence -> pluripotency. Fixed (line 124).
- Line 149: is it U0126 or UO126 like in Figure 2D. Fixed in figure 2.
- Line 294: There is no Figure 7Bii -> Figure S7Bii Fixed, now S11 (line 315).
- Line 409 and 411: Figure S7 Fixed (lines 442 and 444).
- Line 446: though -> thought Removed during sentence rephrasing.
- Line 524: For data in Fig. 1D -> it seems to be wrong as Fig 1D is about apoptosis and not about phosphorylation of signalling pathway. Is it related to Fig2D? Yes, fixed.
- Line 563: The link to the R code did not work on the PDF, so I could not check how data were analysed. See above explanation about moving data to Zenodo. Current data and code is linked on line 646.
- Figure 1F: increase the gene label font or add it to each panel. It is difficult follow the figure. Increased label size.
- Figure 3B to E: Could you indicate the models on top of panels (ii) and (iii). This information is only available in Figure S4. Fixed.

Reviewer 2 Advance Summary and Potential Significance to Field:

In the manuscript entitled 'Signaling Pathway Crosstalk Stimulated by L-Proline Drives

© 2023. Published by The Company of Biologists under the terms of the Creative Commons Attribution License (https://creativecommons.org/licenses/by/4.0/).

Differentiation…', Glover and coworkers confirmed and extended previous observations that the availability of proline modulates different signaling pathways in ESCs. They used an innovative approach to understand the pathways that control proline-induced effects (including morphological transition, cell proliferation/apoptosis, and expression of a few marker genes), and determined whether these pathways act antagonistically or synergistically. The results are very interesting and provide insights into the complexity of the early phase of the developmental process. However, in my opinion, prior to publication, it is necessary to make some clarifications/revisions regarding the terminology used to describe the effects of proline supplementation on ESC behavior.

Reviewer 2 Comments for the Author:

Major points:

- **differentiation vs naïve to early primed transition.**
  In some passages of this manuscript the authors claim that proline supplementation induces ESC differentiation:
  - Thank you for your comments on this manuscript. We appreciate that there is a nuance to the definition of 'differentiation'. On writing this manuscript we considered the transition along the pluripotency to be a form of differentiation as they represent temporally distinct cell populations in vivo. We understand the concerns the reviewer has with the use of this word as the cells in this study have not undergone lineage restriction. We have changed all relevant references to 'differentiation' to either 'pluripotency state transition' or 'transition to primed pluripotency' throughout the manuscript. For methodology-based sentences we have changed the text to 'x days in culture' or similar. The title was changed to 'Signalling pathway crosstalk stimulated by L-proline drives mouse embryonic stem cells to primitive-ectoderm-like cells'. We hope these changes satisfy all the reviewer's comments (below) with respect to this issue

Title

- ….drives differentiation of mouse embryonic stem cells to primitive-ectoderm-like cells
  Changed

Abstract

- sentence 'and each inhibitor blocked specific aspects of differentiation'. Changed on line 20.

Introduction

- pag 2. line 38: sentence 'stimulates differentiation to a second pluripotent population known as early primitive ectoderm-like cells (EPL cells)'. Changed on line 38.
- pag. 2 line 55: sentence 'The continued presence of L-proline in culture then drives EPL cells to neural cells by a series of embryologically relevant intermediate cell types'. Rathjen and co-workers demonstrated that EPL cells are more prone to differentiate (doi: 10.1242/dev.129.11.2649). Proline, on the other hand, does not induce neural differentiation. It is worth noting that recent reports indicate that 3D aggregates of EPL/PiCs elongate earlier than aggregates of naïve cells in gastruloid formation assays (Cermola et al. 2021, doi: 10.1016/j.stemcr.2020.12.013).
  - This paper is intended to show how the L-proline influences cell state during pluripotency. This sentence was included to provide additional context about the established role for L-proline beyond pluripotency and during embryo development. We acknowledge that Rathjen et al. 2002, shows a propensity to differentiate, and this was further demonstrated in Shparberg et al. 2019. We currently are drafting another additional manuscript showing this further. This differentiation lies outside the scope of this manuscript but we expect some of the signalling pathways we show are involved here along the pluripotency continuum will continue to be mechanisms important for differentiation to neurons. We have rephrased this sentence and included the Cermola reference. This is on lines 54-57.
- pag.3 line 58:. sentence. 'L-proline stimulates development/differentiation include..'
  Revised phrasing on lines 54-57.
- pag.3 line 62:..sentence…. 'that drive developmental progression and differentiation..
  Revised to remove 'and differentition' - Line 59-60.

Results

© 2023. Published by The Company of Biologists under the terms of the Creative Commons Attribution License (https://creativecommons.org/licenses/by/4.0/).

- pag. 4 line 93: sentence ..'and then directed to differentiate into EPL cells by addition of 400 μM L- proline for 6 days'. Changed on line 92.
- pag. 4 line 101: sentence ..' at days 2, 4 and 6 of differentiation…' Changed on line 100.
- pag. 4 line 111: sentence ..' After 6 days of differentiation…' it would be better to use 'After 6 days of incubation' Changed on line 109 to '6 days in culture'
- pag. 6 line 167: sentence. …'was designed for the differentiation of ESCs to EPL' Changed on line 124.
- pag. 9 line 286: sentence. …' After 6 days of differentiation…' Changed on line 308.

Discussion

- pag. 10 line 301: sentence ..' during ESC differentiation to EPL cells' Changed on line 322.
- pag. 10 line 303: sentence ..' that mediate self-renewal, differentiation,' Changed on line 324.
- pag. 10 line 309: sentence ..' associated with proline-mediated differentiation to EPL cells,' Changed on line 330.
- pag. 10 line 315: sentence ..' the course of differentiation, Fgfr phosphorylation increased,' Changed on line 336.
- pag. 11 line 337: sentence ..' during ESC differentiation to EPL cells,' Changed on line 357.
- pag. 12 line 366: sentence ..' L-proline in promoting directed differentiation,' Changed on line 387.
- pag. 13 line 392: sentence ..' in differentiation to EPL cells,' Changed on lines 421-422.
- pag. 14 line 447: sentence ..' mechanisms of L-proline-mediated differentiation…,' Revised to remove 'differentiation' - Lines 478.
- pag. 14 line 456: sentence ..' differentiation of pluripotent cells towards neuroectoderm…,' We have retained this since it pertains to post-pluripotency differentiation associated with lineage restriction.
- pag. 15 line 458: sentence ..' proline mediated differentiation provides a useful model…, Changed on line 492-493.

Methods

- pag.15 line 470: sentence 'ESCs were differentiated to EPL Changed on line 503.
- pag.15 line 481: sentence 'At days 2, 4 and 6, differentiating ESCs treated with 1000 u/mL LIF or 330 U/mL LIF,'
- When cultured with LIF, the cells are unable to differentiate. It is worth noting that EPL cells are dependent on LIF for their survival and proliferation.
  - Yes this is correct. We wanted to establish the both 330 and 1000 U/mL LIF are sufficient to keep cells as undifferentiated naïve ESCs (Fig. 1F). We only see the transition to EPL cells when 400 μM L-proline was added to 330 U/mL LIF. We changed the 'differentiation' to 'cultured' on line 514.

Figure legends

- pag. 21 line 660: sentence 'control different aspects of differentiation..' In other passages the authors claim that proline supplementation induces a pluripotency state transition: This has been revised to 'control different aspects of cell state transition' - line 715.

Abstract

- sentence 'drives mouse embryonic stem cells (ESCs) to a transcriptionally distinct pluripotent cell population - early primitive ectoderm-like (EPL) cells - which lies between the naïve and primed states' We have revised this to 'which represents a primed or partially primed pluripotent state'.

Introduction

- pag. 2 line 42. sentence 'EPL cells/PiCs are metastable, as they revert to naïve ESCs upon removal of L-proline'. It is known that differentiated cells are unable to revert spontaneously to the naïve state of pluripotency.

© 2023. Published by The Company of Biologists under the terms of the Creative Commons Attribution License (https://creativecommons.org/licenses/by/4.0/).

EPL cells generated from L-proline and MEDII conditioned media have previously been shown to be metastable (Casalino et al. 2011 and Lake et al. 2000), which is able to occur as they have not fully undergone a lineage restriction. These cells return to a naïve state (ie similar to culture in 1000 U/mL LIF). They do not revert to the ground state (ie 2i culture). As mentioned above, EPL cells have not exited pluripotency but rather represent a primed or partially primed pluripotent cell population (Lines 11, 49 and 486).

- pag. 2 line 48. sentence 'EPL cells are more primed to differentiate than ESCs'. Changed in line with above. Lines 48-49.

Results

- pag. 4 line 115. sentence ….'indicating maintenance of pluripotency'. Previous studies have demonstrated that EPL/PiCs cells generated in DMEM/FBS/LIF medium supplemented with proline are pluripotent stem cells. Evidence from both phenotypic (blastocyst injection/chimera, teratoma, and gastruloid formation), and molecular (transcriptome and global methylation profiling) assays, support the idea that a high proline regimen drives naïve ESCs towards an intermediate/early primed transitional state between naïve and primed pluripotency states. Moreover, EPL/PiCs cells have been shown to be pluripotent undifferentiated cells (doi: 10.3390/CELLS11142125) and competent at primordial germ cell differentiation (Cermola et al. 2021, doi: 10.1016/j.stemcr.2020.12.013). Notably, EPL cells are capable of spontaneously reverting to the naïve state, while differentiated cells are unable to do so. In my opinion, proline cannot be considered an inducer of ESC differentiation. Moreover, the resulting EPL/PiCs cells, which are captured in a stable intermediate state of pluripotency, cannot be defined as differentiated cells.
    - We agree that, based on our results, there is no reason to believe EPL cells have undergone lineage restriction and have changed the wording accordingly throughout the manuscript. L- proline does drive ESCs to neurectoderm and mature neurons as mentioned above and in Shparberg et al. 2019 and Rathjen et al. 2002). We have also clarified the position of L-proline as a primed or partially primed cell population at several points in the manuscript (Lines 11, 49 and 486).

2) the role played by Snat2

- In the schemes shown in Fig. 2 and Fig. 5, the modulation of Stat, mTor, Pi3K, and Mek signaling pathways by a high proline regimen is dependent on proline uptake through Snat2. However, no experimental evidence regarding the role of this specific amino acid channel in proline-mediated modulation of the signaling pathways is presented here. Nevertheless, it is highly plausible that this is the case.
    - L-proline has been shown to enter the cell via Snat2 in stem cells to alter phosphorylation of signalling pathways (Tan et al. 2011). This information and reference are included on line 58. We included Snat2 in the introduction and in these figures to provide context, but study of SNAT2 is outside the scope of this study.

Minor points:

- The AAR pathway was investigated through transcriptome analysis, in combination with pharmacological (specific inhibitor of the prolyl-tRNA synthetase, Halofuginone) and genetic approaches (downregulation and ectopic expression of ATF4). The results obtained supported the idea that proline supplementation induces alleviation of the amino acid stress response (AAR) pathway, specifically the GCN2-ATF4 signaling pathway (D'Aniello et al., 2015, DOI: 10.1038/cdd.2015.24).
- The TGF-ß pathway has been shown to play a role in proline-induced primed states via autocrine FGF and TGF-β signaling pathways. Supplemental proline was found to induce Smad2 phosphorylation, as reported by D'Aniello et al. in 2017 (doi: 10.1016/j.stemcr.2016.11.011). Additionally, the presence of the TGF-ß chemical inhibitor SB431542 impaired proline-induced phenotypic transition (D'Aniello et al., 2017, doi: 10.1016/j.stemcr.2016.11.011).
    - Thank you for this comment. We are aware that there are several additional mechanisms which are activated in response to L-proline supplementation including alteration of the AAR, changes in cellular metabolism, epigenetic changes, and other signalling pathways. Signalling is an important piece of this equation and the inhibitors chosen were based on the best evidence available when they study was

© 2023. Published by The Company of Biologists under the terms of the Creative Commons Attribution License (https://creativecommons.org/licenses/by/4.0/).

conducted, while keeping the number of combinations within a reasonable scope. We have indicated in the discussion that TGF-β has been identified as a signalling pathway activated by L-proline (Line 414), and this is a pathway are actively studying with mass spectrometry-based phosphorylation analysis which allows for more high-throughput study of the cell signalling. Nonetheless, we believe the current work still provides robust scientific progress in amino acid regulated signalling, signalling pathway cross talk and the pluripotency continuum.

Second decision letter

MS ID#: DEVELOP/2023/201704

MS TITLE: Signalling pathway crosstalk stimulated by L-proline drives mouse embryonic stem cells to primitive-ectoderm-like cells

AUTHORS: Hannah J Glover, Holly Holliday, Rachel A Shparberg, David Winkler, Margot Day, and Michael B Morris

I have now received all the referees reports on the above manuscript, and have reached a decision. The referees' comments are appended below, or you can access them online: please go to BenchPress and click on the 'Manuscripts with Decisions' queue in the Author Area.

The overall evaluation is positive and we would like to publish a revised manuscript in Development, provided that the referees' comments can be satisfactorily addressed. Please attend to all of the reviewers' comments in your revised manuscript and detail them in your point-by-point response. If you do not agree with any of their criticisms or suggestions explain clearly why this is so. If it would be helpful, you are welcome to contact us to discuss your revision in greater detail. Please send us a point-by-point response indicating your plans for addressing the referees comments, and we will look over this and provide further guidance.

Reviewer 1

Advance summary and potential significance to field

Glover and colleagues analysed the signalling pathways underpinning the pluripotency continuum from naïve ESC to EPL to peri-gastrulation states (by forming embryoid bodies) and more generally to the role of L-Proline in this conversion.
The authors characterised the signalling pathways involved in the maintenance of EPL cells. They have done so by performing comprehensive study on phosphorylation of 5 pathways across time, and by perturbing the pathways using inhibitors.
There is an important body of work including many western blots, qRT-PCR and image analysis, which are appropriate read-outs for this study. Computational analyses unveil relationships among the signalling pathways scrutinised in the study. Novel insights could be gleaned regarding the dynamic and synergy of these pathways during pluripotency dismantling and of the role of L-Proline in pluripotency maintenance, which could be of interest to stem cell biologists.

Comments for the author

The authors have successfully addressed the issues raised in the first round of review.

There are only two minor edits:
> Figure S1 and S7 are of very low quality which makes them almost impossible to read.
> The explanation about the bands is a great addition. It is totally appreciated that antibodies are not 100% accurate and there is no issues withe this. So, in order to add the maximum of transparency about data collection, it would be nice to show which band was selected for ALL antibodies. This could also help future work on these antibodies.

© 2023. Published by The Company of Biologists under the terms of the Creative Commons Attribution License (https://creativecommons.org/licenses/by/4.0/).

Reviewer 2

Advance summary and potential significance to field

In the manuscript entitled 'Signaling Pathway Crosstalk Stimulated by L-Proline Drives Differentiation…', Glover and coworkers confirmed and extended previous observations that the availability of proline modulates different signaling pathways in ESCs. They used an innovative approach to understand the pathways that control proline-induced effects (including morphological transition, cell proliferation/apoptosis, and expression of a few marker genes), and determined whether these pathways act antagonistically or synergistically. The results are very interesting and provide insights into the complexity of the early phase of the developmental process.

Comments for the author

The authors have addressed all the issues rasied by this reviewer.

Second revision

Author response to reviewers' comments

Dear Editors and Reviewers,

Thank you for reviewing this manuscript. We appreciate your valuable feedback and are excited to have our work published in *Development*. We have revised the manuscript to include the minor changes suggested:

1. Figure S1 and S7 are of very low quality which makes them almost impossible to read. We have provided the figures at in high resolution both as a word document and a pdf.
2. The explanation about the bands is a great addition. It is totally appreciated that antibodies are not 100% accurate and there is no issues with this. So, in order to add the maximum of transparency about data collection, it would be nice to show which band was selected for ALL antibodies. This could also help future work on these antibodies. We have expanded this figure to include examples for all antibodies used in this study. For additional clarity, we have made minor changes to the methods section (Line 570) and the figure legend to better represent the figure.

We believe these changes address the concerns raised by the reviewers. Thank you for your time and comments.

Sincerely,
Dr. Hannah Glover and Dr. Michael Morris, on behalf of all authors

---

Third decision letter

MS ID#: DEVELOP/2023/201704

MS TITLE: Signalling pathway crosstalk stimulated by L-proline drives mouse embryonic stem cells to primitive-ectoderm-like cells

AUTHORS: Hannah J Glover, Holly Holliday, Rachel A Shparberg, David Winkler, Margot Day, and Michael B Morris

© 2023. Published by The Company of Biologists under the terms of the Creative Commons Attribution License (https://creativecommons.org/licenses/by/4.0/).

ARTICLE TYPE: Research Article

I am happy to tell you that your manuscript has been accepted for publication in Development, pending our standard ethics checks

© 2023. Published by The Company of Biologists under the terms of the Creative Commons Attribution License (https://creativecommons.org/licenses/by/4.0/).
